# Supplementary material for: Unusual Square Pyramidal Chalcogenide Mo5 Cluster with Bridging Pyrazolate-Ligands
Source: Int J Mol Sci. 2023 Feb 8;24(4):3440. doi: 10.3390/ijms24043440 (PMC9962213; doi:10.3390/ijms24043440)

checkCIF/PLATON report

Structure factors have been supplied for datablock(s) jul086

THIS REPORT IS FOR GUIDANCE ONLY. IF USED AS PART OF A REVIEW PROCEDURE FOR PUBLICATION, IT SHOULD NOT REPLACE THE EXPERTISE OF AN EXPERIENCED CRYSTALLOGRAPHIC REFEREE.

No syntax errors found.   CIF dictionary   Interpreting this report

Datablock: jul086

Bond precision: C-C = 0.0151 A   Wavelength=0.71073

Cell:	a=15.632(1)	b=25.5634(15)	c=12.2766(8)
	alpha=90	beta=90	gamma=90
Temperature:	150 K		

	Calculated	Reported
Volume	4905.8(5)	4905.8(5)
Space group	C 2 2 21	C 2 2 21
Hall group	C 2c 2	C 2c 2
Moiety formula	4(C27 H28 Mo5 N18 Se5), 3(O2), 8(Br), 2(O)	C27 H32 Mo5 N18 Se5, 2(Br), 2(H2 O)
Sum formula	C108 H112 Br8 Mo20 N72 O8 Se20	C27 H36 Br2 Mo5 N18 O2 Se5
Mr	6683.90	1679.06
Dx,g cm-3	2.262	2.273
Z	1	4
Mu (mm-1)	6.629	6.629
F000	3128.0	3160.0
F000'	3090.39	
h,k,lmax	20,32,15	20,32,15
Nref	5505[3043]	5450
Tmin,Tmax	0.315,0.451	0.519,0.746
Tmin'	0.239	

Correction method= # Reported T Limits: Tmin=0.519 Tmax=0.746
AbsCorr = MULTI-SCAN

Data completeness= 1.79/0.99   Theta(max)= 27.256

R(reflections)= 0.0331(4676)   wR2(reflections)= 0.0852(5450)

S = 1.064   Npar= 293

The following ALERTS were generated. Each ALERT has the format
test-name_ALERT_alert-type_alert-level.
Click on the hyperlinks for more details of the test.

Alert level C

```
STRVA01_ALERT_4_C          Flack test results are ambiguous.
           From the CIF: _refine_ls_abs_structure_Flack    0.423
           From the CIF: _refine_ls_abs_structure_Flack_su    0.011
PLAT041_ALERT_1_C Calc. and Reported SumFormula    Strings  Differ     Please Check
PLAT042_ALERT_1_C Calc. and Reported MoietyFormula Strings  Differ     Please Check
PLAT043_ALERT_1_C Calculated and Reported Mol. Weight Differ by ..      32.34 Check
PLAT068_ALERT_1_C Reported F000 Differs from Calcd (or Missing)...     Please Check
PLAT260_ALERT_2_C Large Average Ueq of Residue Including       O3W      0.118 Check
PLAT260_ALERT_2_C Large Average Ueq of Residue Including       O2W      0.118 Check
PLAT342_ALERT_3_C Low Bond Precision on  C-C Bonds ...............    0.01512 Ang.
PLAT601_ALERT_2_C Unit Cell Contains Solvent Accessible VOIDS of .         34 Ang**3
PLAT911_ALERT_3_C Missing FCF Refl Between Thmin & STh/L=    0.600          3 Report
```

Alert level G

```
FORMU01_ALERT_2_G  There is a discrepancy between the atom counts in the
            _chemical_formula_sum and the formula from the _atom_site* data.
            Atom count from _chemical_formula_sum:C27 H36 Br2 Mo5 N18 O2 Se5
            Atom count from the _atom_site data:  C27 H28 Br2 Mo5 N18 O2 Se5
CELLZ01_ALERT_1_G Difference between formula and atom_site contents detected.
CELLZ01_ALERT_1_G WARNING: H atoms missing from atom site list. Is this intentional?
           From the CIF: _cell_formula_units_Z    4
           From the CIF: _chemical_formula_sum  C27 H36 Br2 Mo5 N18 O2 Se5
           TEST: Compare cell contents of formula and atom_site data

           atom    Z*formula  cif sites diff
           C         108.00    108.00    0.00
           H         144.00    112.00   32.00
           Br          8.00      8.00    0.00
           Mo         20.00     20.00    0.00
           N          72.00     72.00    0.00
           O           8.00      8.00    0.00
           Se         20.00     20.00    0.00
PLAT003_ALERT_2_G Number of Uiso or Uij Restrained non-H Atoms ...          2 Report
PLAT007_ALERT_5_G Number of Unrefined Donor-H Atoms .............          2 Report
PLAT033_ALERT_4_G Flack x Value Deviates > 3.0 * sigma from Zero .      0.423 Note
PLAT045_ALERT_1_G Calculated and Reported Z Differ by a Factor ...      0.250 Check
PLAT171_ALERT_4_G The CIF-Embedded .res File Contains EADP Records          2 Report
PLAT186_ALERT_4_G The CIF-Embedded .res File Contains ISOR Records          1 Report
PLAT300_ALERT_4_G Atom Site Occupancy of N12        Constrained at        0.5 Check
PLAT300_ALERT_4_G Atom Site Occupancy of C13        Constrained at        0.5 Check
PLAT300_ALERT_4_G Atom Site Occupancy of C14        Constrained at        0.5 Check
PLAT300_ALERT_4_G Atom Site Occupancy of C15        Constrained at        0.5 Check
PLAT300_ALERT_4_G Atom Site Occupancy of O1W        Constrained at        0.5 Check
PLAT300_ALERT_4_G Atom Site Occupancy of O3W        Constrained at       0.25 Check
```

```
PLAT300_ALERT_4_G Atom Site Occupancy of O2W        Constrained at       0.25 Check
PLAT301_ALERT_3_G Main Residue  Disorder .............(Resd  1  )          7% Note
PLAT302_ALERT_4_G Anion/Solvent/Minor-Residue Disorder (Resd  2  )       100% Note
PLAT302_ALERT_4_G Anion/Solvent/Minor-Residue Disorder (Resd  3  )       100% Note
PLAT302_ALERT_4_G Anion/Solvent/Minor-Residue Disorder (Resd  5  )       100% Note
PLAT304_ALERT_4_G Non-Integer Number of Atoms in ..... (Resd  3  )       0.50 Check
PLAT304_ALERT_4_G Non-Integer Number of Atoms in ..... (Resd  5  )       0.25 Check
PLAT311_ALERT_2_G Isolated Disordered Oxygen Atom (No H's ?) .....        O1W Check
PLAT311_ALERT_2_G Isolated Disordered Oxygen Atom (No H's ?) .....        O2W Check
PLAT764_ALERT_4_G Overcomplete CIF Bond List Detected (Rep/Expd) .       1.22 Ratio
PLAT773_ALERT_2_G Check long C-C Bond in CIF: C15      --C15             1.81 Ang.
PLAT773_ALERT_2_G Check long C-C Bond in CIF: C15      --C14             1.83 Ang.
PLAT773_ALERT_2_G Check long C-C Bond in CIF: C13      --C13             1.95 Ang.
PLAT779_ALERT_4_G Suspect or Irrelevant (Bond) Angle(s) in CIF ...      15.10 Deg.
              C15  -N11  -N12     4_555   1_555   1_555 ......     #  122 Check
PLAT779_ALERT_4_G Suspect or Irrelevant (Bond) Angle(s) in CIF ...      15.10 Deg.
              C15  -N11  -N12     1_555   1_555   4_555 ......     #  125 Check
PLAT779_ALERT_4_G Suspect or Irrelevant (Bond) Angle(s) in CIF ...      34.00 Deg.
              C15  -N12  -C14     4_555   1_555   4_555 ......     #  134 Check
PLAT779_ALERT_4_G Suspect or Irrelevant (Bond) Angle(s) in CIF ...      19.20 Deg.
              C13  -N12  -C14     1_555   1_555   4_555 ......     #  135 Check
PLAT779_ALERT_4_G Suspect or Irrelevant (Bond) Angle(s) in CIF ...      25.90 Deg.
              C13  -C15  -C14     4_555   1_555   1_555 ......     #  141 Check
PLAT779_ALERT_4_G Suspect or Irrelevant (Bond) Angle(s) in CIF ...      44.10 Deg.
              N11  -C15  -C15     1_555   1_555   4_555 ......     #  145 Check
PLAT779_ALERT_4_G Suspect or Irrelevant (Bond) Angle(s) in CIF ...      20.80 Deg.
              C14  -C15  -C14     1_555   1_555   4_555 ......     #  150 Check
PLAT779_ALERT_4_G Suspect or Irrelevant (Bond) Angle(s) in CIF ...       6.00 Deg.
              C14  -C14  -C13     4_555   1_555   1_555 ......     #  154 Check
PLAT779_ALERT_4_G Suspect or Irrelevant (Bond) Angle(s) in CIF ...       9.00 Deg.
              C15  -C14  -N12     1_555   1_555   4_555 ......     #  161 Check
PLAT779_ALERT_4_G Suspect or Irrelevant (Bond) Angle(s) in CIF ...      41.00 Deg.
              C13  -C14  -C15     1_555   1_555   4_555 ......     #  164 Check
PLAT779_ALERT_4_G Suspect or Irrelevant (Bond) Angle(s) in CIF ...       7.00 Deg.
              C14  -C13  -C14     4_555   1_555   1_555 ......     #  228 Check
PLAT779_ALERT_4_G Suspect or Irrelevant (Bond) Angle(s) in CIF ...      16.00 Deg.
              C15  -C13  -N12     4_555   1_555   1_555 ......     #  231 Check
PLAT779_ALERT_4_G Suspect or Irrelevant (Bond) Angle(s) in CIF ...      13.00 Deg.
              C14  -C13  -C13     4_555   1_555   4_555 ......     #  233 Check
PLAT779_ALERT_4_G Suspect or Irrelevant (Bond) Angle(s) in CIF ...       6.00 Deg.
              C14  -C13  -C13     1_555   1_555   4_555 ......     #  235 Check
PLAT860_ALERT_3_G Number of Least-Squares Restraints ............          12 Note
PLAT898_ALERT_4_G Second Reported H-M Symbol in CIF Ignored ......          ! Check
PLAT910_ALERT_3_G Missing # of FCF Reflection(s) Below Theta(Min).          2 Note
PLAT912_ALERT_4_G Missing # of FCF Reflections Above STh/L=  0.600         17 Note
PLAT933_ALERT_2_G Number of HKL-OMIT Records in Embedded .res File          4 Note
PLAT961_ALERT_5_G Dataset Contains no Negative Intensities .......     Please Check
PLAT978_ALERT_2_G Number C-C Bonds with Positive Residual Density.          0 Info
```

---

0 **ALERT level A** = Most likely a serious problem - resolve or explain
0 **ALERT level B** = A potentially serious problem, consider carefully
10 **ALERT level C** = Check. Ensure it is not caused by an omission or oversight
49 **ALERT level G** = General information/check it is not something unexpected

7 ALERT type 1 CIF construction/syntax error, inconsistent or missing data

```
12 ALERT type 2 Indicator that the structure model may be wrong or deficient
 5 ALERT type 3 Indicator that the structure quality may be low
33 ALERT type 4 Improvement, methodology, query or suggestion
 2 ALERT type 5 Informative message, check
```

It is advisable to attempt to resolve as many as possible of the alerts in all categories. Often the minor alerts point to easily fixed oversights, errors and omissions in your CIF or refinement strategy, so attention to these fine details can be worthwhile. In order to resolve some of the more serious problems it may be necessary to carry out additional measurements or structure refinements. However, the purpose of your study may justify the reported deviations and the more serious of these should normally be commented upon in the discussion or experimental section of a paper or in the "special_details" fields of the CIF. checkCIF was carefully designed to identify outliers and unusual parameters, but every test has its limitations and alerts that are not important in a particular case may appear. Conversely, the absence of alerts does not guarantee there are no aspects of the results needing attention. It is up to the individual to critically assess their own results and, if necessary, seek expert advice.

Publication of your CIF in IUCr journals

A basic structural check has been run on your CIF. These basic checks will be run on all CIFs submitted for publication in IUCr journals (*Acta Crystallographica*, *Journal of Applied Crystallography*, *Journal of Synchrotron Radiation*); however, if you intend to submit to *Acta Crystallographica Section C* or *E* or *IUCrData*, you should make sure that full publication checks are run on the final version of your CIF prior to submission.

Publication of your CIF in other journals

Please refer to the *Notes for Authors* of the relevant journal for any special instructions relating to CIF submission.

PLATON version of 12/09/2022; check.def file version of 09/08/2022

Datablock jul086 - ellipsoid plot

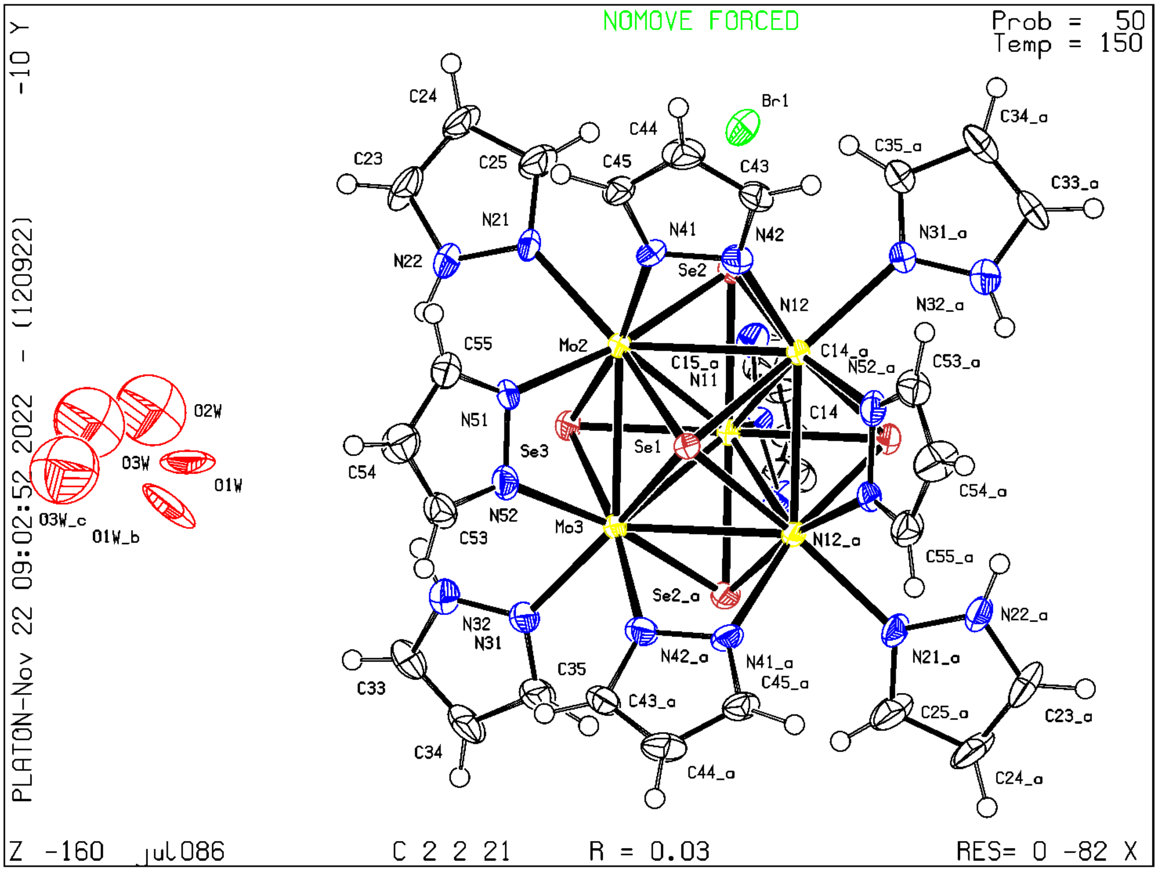

Supplement: Supplementary file 1 [file ijms-24-03440-s001.zip › checkcif_Mo5ox.pdf]
