# Supplementary material for: Unusual Square Pyramidal Chalcogenide Mo5 Cluster with Bridging Pyrazolate-Ligands
Source: Int J Mol Sci. 2023 Feb 8;24(4):3440. doi: 10.3390/ijms24043440 (PMC9962213; doi:10.3390/ijms24043440)

checkCIF/PLATON report

Structure factors have been supplied for datablock(s) jul081_3

THIS REPORT IS FOR GUIDANCE ONLY. IF USED AS PART OF A REVIEW PROCEDURE FOR PUBLICATION, IT SHOULD NOT REPLACE THE EXPERTISE OF AN EXPERIENCED CRYSTALLOGRAPHIC REFEREE.

No syntax errors found. CIF dictionary Interpreting this report

Datablock: jul081_3

Bond precision:	C-C = 0.0038 A		Wavelength=0.71073
Cell:	a=15.6443(4)	b=15.6443(4)	c=11.3058(2)
	alpha=90	beta=90	gamma=90
Temperature:	150 K		

	Calculated	Reported
Volume	2767.03(15)	2767.03(15)
Space group	P 4/n	P 4/n
Hall group	-P 4a	-P 4a
Moiety formula	C25 H28 Mo5 N18 Se5, 4(C3 H4 N2), Br, 2(C)	C27 H32 Mo5 N18 Se5, 4(C3 H4 N2), Br
Sum formula	C39 H44 Br Mo5 N26 Se5	C39 H48 Br Mo5 N26 Se5
Mr	1831.40	1835.44
Dx,g cm-3	2.198	2.203
Z	2	2
Mu (mm-1)	5.171	5.171
F000	1750.0	1758.0
F000’	1731.66	
h,k,lmax	20,20,15	20,20,15
Nref	3442	3441
Tmin,Tmax	0.410,0.538	0.574,0.746
Tmin’	0.308	

Correction method= # Reported T Limits: Tmin=0.574 Tmax=0.746
AbsCorr = MULTI-SCAN

Data completeness= 1.000 Theta(max)= 28.298

R(reflections)= 0.0197(3142) wR2(reflections)= 0.0506(3441)

S = 1.053 Npar= 183

The following ALERTS were generated. Each ALERT has the format
test-name_ALERT_alert-type_alert-level.
Click on the hyperlinks for more details of the test.

Alert level C

```
PLAT041_ALERT_1_C Calc. and Reported SumFormula    Strings  Differ     Please Check
PLAT042_ALERT_1_C Calc. and Reported MoietyFormula Strings  Differ     Please Check
PLAT043_ALERT_1_C Calculated and Reported Mol. Weight Differ by ..       4.04 Check
PLAT068_ALERT_1_C Reported F000 Differs from Calcd (or Missing)...     Please Check
PLAT972_ALERT_2_C Check Calcd Resid. Dens.  0.70Ang From Br1            -1.61 eA-3
```

Alert level G

```
FORMU01_ALERT_2_G  There is a discrepancy between the atom counts in the
            _chemical_formula_sum and the formula from the _atom_site* data.
            Atom count from _chemical_formula_sum:C39 H48 Br1 Mo5 N26 Se5
            Atom count from the _atom_site data:  C39 H44 Br1 Mo5 N26 Se5
CELLZ01_ALERT_1_G Difference between formula and atom_site contents detected.
CELLZ01_ALERT_1_G WARNING: H atoms missing from atom site list. Is this intentional?
           From the CIF: _cell_formula_units_Z    2
           From the CIF: _chemical_formula_sum  C39 H48 Br Mo5 N26 Se5
           TEST: Compare cell contents of formula and atom_site data

           atom    Z*formula  cif sites diff
           C         78.00     78.00    0.00
           H         96.00     88.00    8.00
           Br         2.00      2.00    0.00
           Mo        10.00     10.00    0.00
           N         52.00     52.00    0.00
           Se        10.00     10.00    0.00
PLAT007_ALERT_5_G Number of Unrefined Donor-H Atoms ..............           2 Report
PLAT168_ALERT_4_G The CIF-Embedded .res File Contains EXYZ Records           1 Report
PLAT171_ALERT_4_G The CIF-Embedded .res File Contains EADP Records           1 Report
PLAT232_ALERT_2_G Hirshfeld Test Diff (M-X)  Mo1      --Se2      .         7.3 s.u.
PLAT232_ALERT_2_G Hirshfeld Test Diff (M-X)  Mo2      --Se1      .        18.0 s.u.
PLAT232_ALERT_2_G Hirshfeld Test Diff (M-X)  Mo2      --Se2_a    .         8.5 s.u.
PLAT300_ALERT_4_G Atom Site Occupancy of N12        Constrained at        0.25 Check
PLAT300_ALERT_4_G Atom Site Occupancy of C12        Constrained at        0.25 Check
PLAT300_ALERT_4_G Atom Site Occupancy of C13        Constrained at         0.5 Check
PLAT301_ALERT_3_G Main Residue  Disorder ..............(Resd  1  )          4% Note
PLAT302_ALERT_4_G Anion/Solvent/Minor-Residue Disorder (Resd  4  )        100% Note
PLAT304_ALERT_4_G Non-Integer Number of Atoms in ..... (Resd  3  )        0.25 Check
PLAT304_ALERT_4_G Non-Integer Number of Atoms in ..... (Resd  4  )        0.50 Check
PLAT432_ALERT_2_G Short Inter X...Y Contact  N11      ..C13      .        2.25 Ang.
                                         1/2-x,1/2-y,z  =      3_555 Check
PLAT432_ALERT_2_G Short Inter X...Y Contact  N11      ..C13      .        2.25 Ang.
                                             y,1/2-x,z  =      4_555 Check
PLAT432_ALERT_2_G Short Inter X...Y Contact  N11      ..C13      .        2.25 Ang.
                                                 x,y,z  =      1_555 Check
PLAT432_ALERT_2_G Short Inter X...Y Contact  N11      ..C13      .        2.25 Ang.
                                             1/2-y,x,z  =      2_555 Check
PLAT432_ALERT_2_G Short Inter X...Y Contact  C13      ..C13      .        0.96 Ang.
                                             y,1/2-x,z  =      4_555 Check
PLAT432_ALERT_2_G Short Inter X...Y Contact  C13      ..C13      .        0.96 Ang.
                                             1/2-y,x,z  =      2_555 Check
```

```
PLAT432_ALERT_2_G Short Inter X...Y Contact  C13      ..C13      .       1.36 Ang.
                                           1/2-x,1/2-y,z  =      3_555 Check
PLAT779_ALERT_4_G Suspect or Irrelevant (Bond) Angle(s) in CIF ...      31.00 Deg.
              C13  -N12  -C13     1_555   1_555   2_555 ......     #   99 Check
PLAT779_ALERT_4_G Suspect or Irrelevant (Bond) Angle(s) in CIF ...      31.00 Deg.
              C13  -N12  -C13     1_555   1_555   4_555 ......     #  103 Check
PLAT779_ALERT_4_G Suspect or Irrelevant (Bond) Angle(s) in CIF ...      43.60 Deg.
              C13  -N12  -C13     2_555   1_555   4_555 ......     #  106 Check
PLAT790_ALERT_4_G Centre of Gravity not Within Unit Cell: Resd.  #          4 Note
              C
PLAT898_ALERT_4_G Second Reported H-M Symbol in CIF Ignored ......          ! Check
PLAT912_ALERT_4_G Missing # of FCF Reflections Above STh/L=  0.600          1 Note
PLAT933_ALERT_2_G Number of HKL-OMIT Records in Embedded .res File          1 Note
PLAT961_ALERT_5_G Dataset Contains no Negative Intensities .......     Please Check
PLAT978_ALERT_2_G Number C-C Bonds with Positive Residual Density.          7 Info
```

---

   0 **ALERT level A** = Most likely a serious problem - resolve or explain
   0 **ALERT level B** = A potentially serious problem, consider carefully
   5 **ALERT level C** = Check. Ensure it is not caused by an omission or oversight
  32 **ALERT level G** = General information/check it is not something unexpected

   6 ALERT type 1 CIF construction/syntax error, inconsistent or missing data
  14 ALERT type 2 Indicator that the structure model may be wrong or deficient
   1 ALERT type 3 Indicator that the structure quality may be low
  14 ALERT type 4 Improvement, methodology, query or suggestion
   2 ALERT type 5 Informative message, check

It is advisable to attempt to resolve as many as possible of the alerts in all categories. Often the minor alerts point to easily fixed oversights, errors and omissions in your CIF or refinement strategy, so attention to these fine details can be worthwhile. In order to resolve some of the more serious problems it may be necessary to carry out additional measurements or structure refinements. However, the purpose of your study may justify the reported deviations and the more serious of these should normally be commented upon in the discussion or experimental section of a paper or in the "special_details" fields of the CIF. checkCIF was carefully designed to identify outliers and unusual parameters, but every test has its limitations and alerts that are not important in a particular case may appear. Conversely, the absence of alerts does not guarantee there are no aspects of the results needing attention. It is up to the individual to critically assess their own results and, if necessary, seek expert advice.

Publication of your CIF in IUCr journals

A basic structural check has been run on your CIF. These basic checks will be run on all CIFs submitted for publication in IUCr journals (*Acta Crystallographica*, *Journal of Applied Crystallography*, *Journal of Synchrotron Radiation*); however, if you intend to submit to *Acta Crystallographica Section C* or *E* or *IUCrData*, you should make sure that full publication checks are run on the final version of your CIF prior to submission.

Publication of your CIF in other journals

Please refer to the *Notes for Authors* of the relevant journal for any special instructions relating to CIF submission.

PLATON version of 12/09/2022; check.def file version of 09/08/2022

Datablock jul081_3 - ellipsoid plot

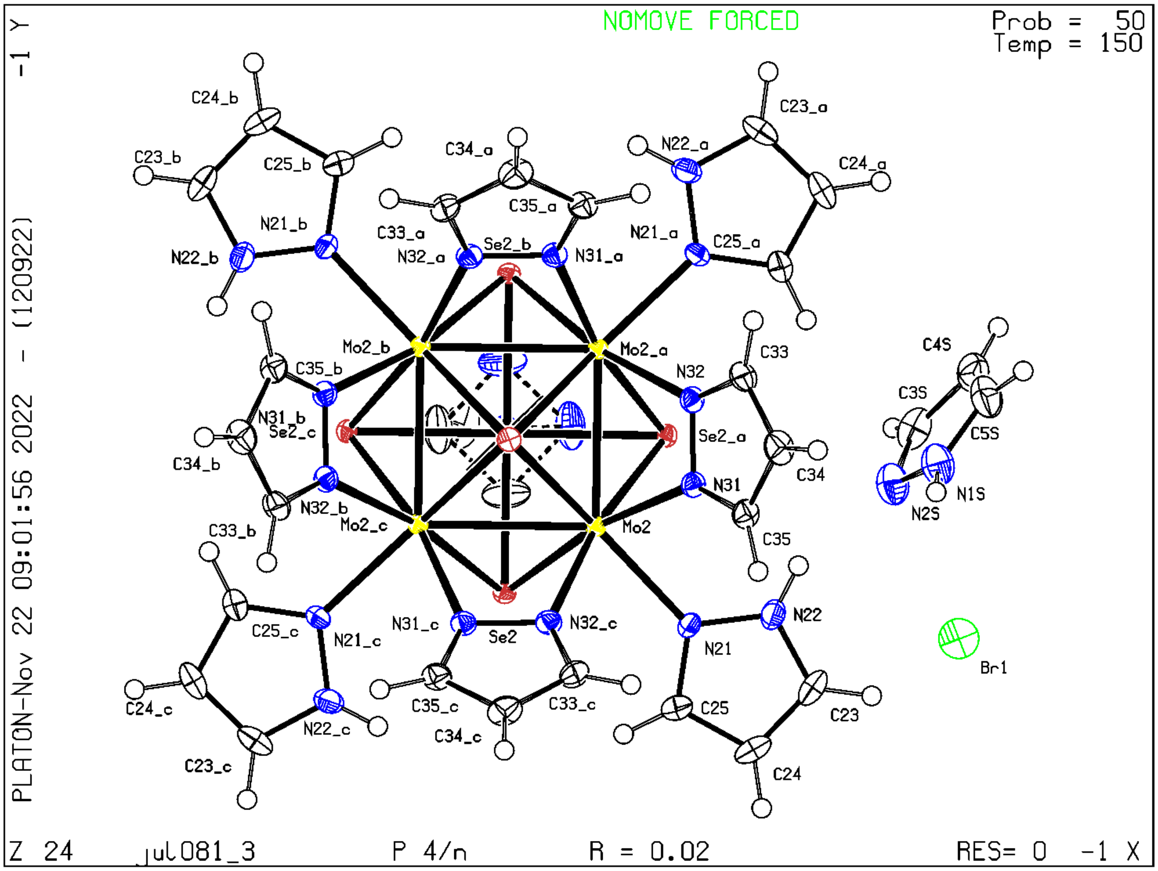

Supplement: Supplementary file 1 [file ijms-24-03440-s001.zip › checkcif_Mo5red.pdf]
