# Supplementary material for: Unusual Square Pyramidal Chalcogenide Mo5 Cluster with Bridging Pyrazolate-Ligands
Source: Int J Mol Sci. 2023 Feb 8;24(4):3440. doi: 10.3390/ijms24043440 (PMC9962213; doi:10.3390/ijms24043440)
Supplement: Supplementary file 1 [file ijms-24-03440-s001.zip › ESI revised.pdf]

Unusual Square Pyramidal Chalcogenide Mo_5 Cluster with Bridging Pyrazolate-Ligands

Iulia V. Savina [1], Anton A. Ivanov [1,*], Darya V. Evtushok [1], Yakov M. Gayfulin [1], Andrey Y. Komarovskikh [1], Mikhail M. Syrokvashin [1], Mariia N. Ivanova [1], Igor P. Asanov [1], Ilia V. Eltsov [2], Natalia V. Kuratieva [1], Yuri V. Mironov [1,*] and Michael A. Shestopalov [1]

[1] Nikolaev Institute of Inorganic Chemistry of Siberian Branch of Russian Academy of Sciences, 3 Acad. Lavrentiev ave., 630090, Novosibirsk, Russia
[2] Department of Natural Sciences, Novosibirsk State University, 1 Pirogova st., 630090, Novosibirsk, Russia
*Correspondence: ivanov338@niic.nsc.ru (A.A.I.), yuri@niic.nsc.ru (Y.V.M.)

TABLE OF CONTENT

Powder X-ray diffraction analysis 3
UV-vis spectroscopy 4
Mo_5red: packing in crystal structure 5
Mo_5ox: packing in crystal structure and powder X-ray diffraction analysis 8
DFT calculations 10
XPS data 13
NMR spectroscopy data 18
Mass-spectrometry data 22
EPR spectroscopy data 23
Cyclic voltammetry data 25
Characterization of compounds: IR spectra, TGA curves, UV-vis spectra and PXRD data 26
Crystal structure data 29

Powder X-ray diffraction analysis

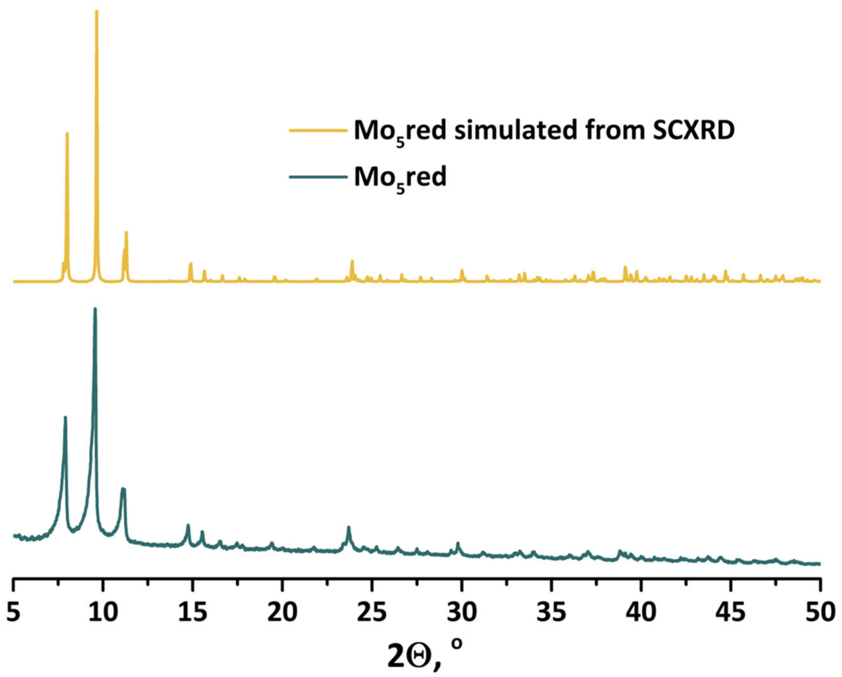

Figure S1. XRPD pattern of **Mo_5red** in comparison with calculated one from the SCXRD data.

UV-vis spectroscopy

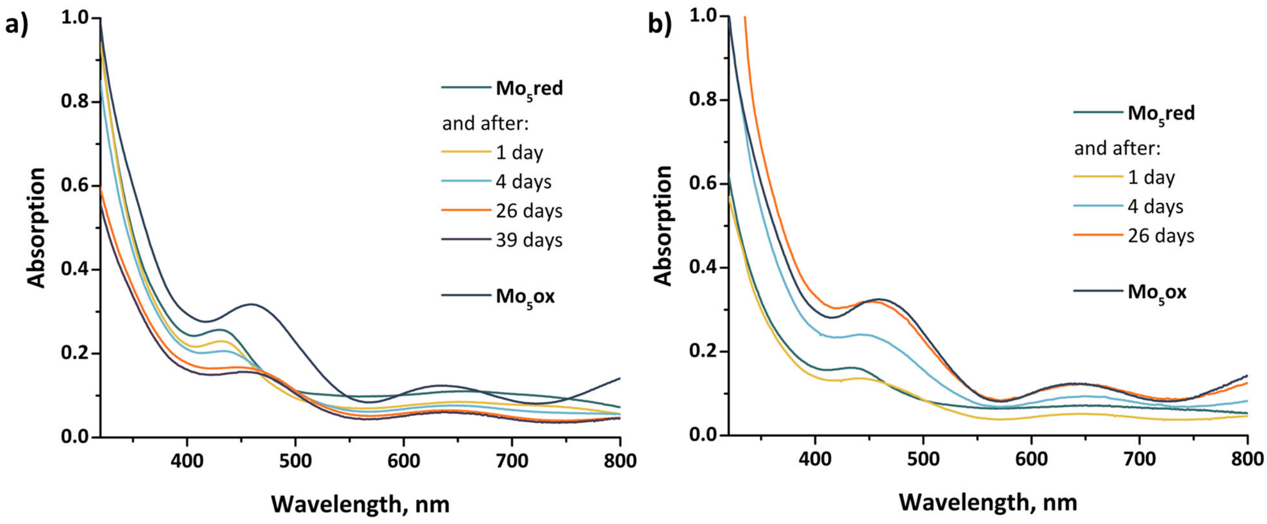

Figure S2. UV-vis spectra of **Mo_5red** acetonitrile (a) or DCM (b) solutions in time in comparison with **Mo_5ox**.

Mo_5red: packing in crystal structure

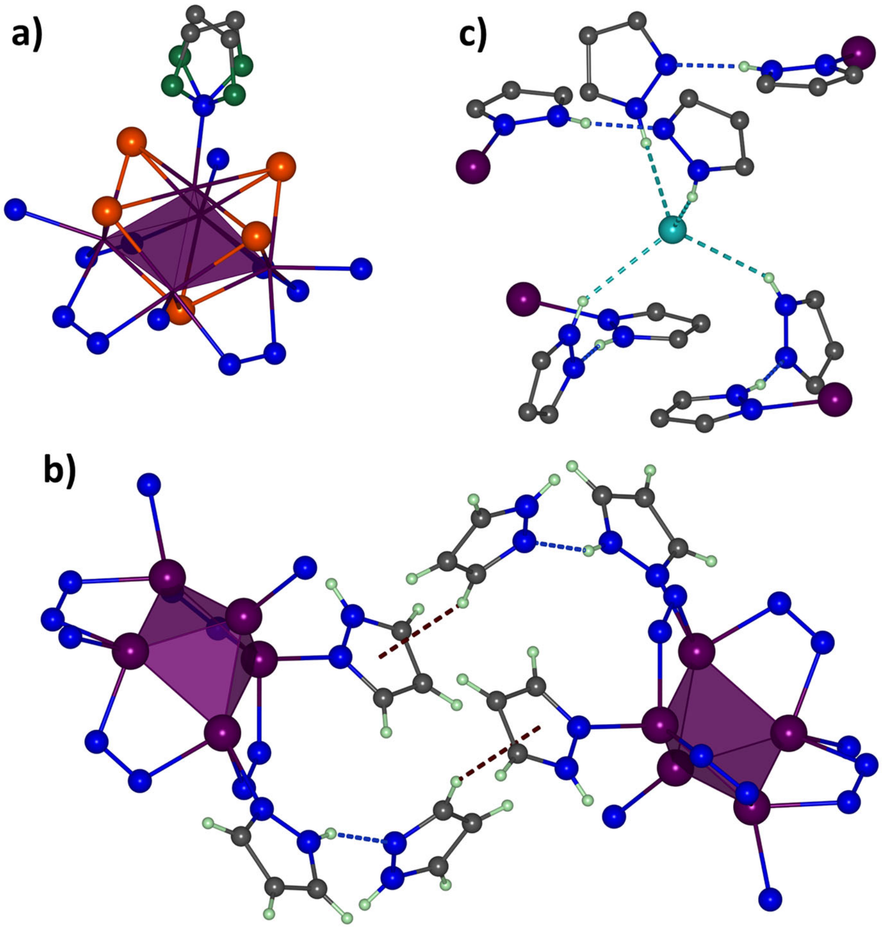

Figure S3. Packing in the crystal structure of **Mo_5red**. a) Disorder of axial pyrazole ligand over four positions. b) Hydrogen bonds and C-H···π stacking interactions between solvated pyrazole molecules and pzH-ligands. c) Hydrogen bonds between bromine-ion and solvated pyrazole molecules. Color code: Mo – violet, Mo_5 – violet square pyramid, C – gray, N – blue, Br – cyan, H – light green, disordered C/N – green, C-H···π – red, N-H···N – blue, N-H···Br – cyan.

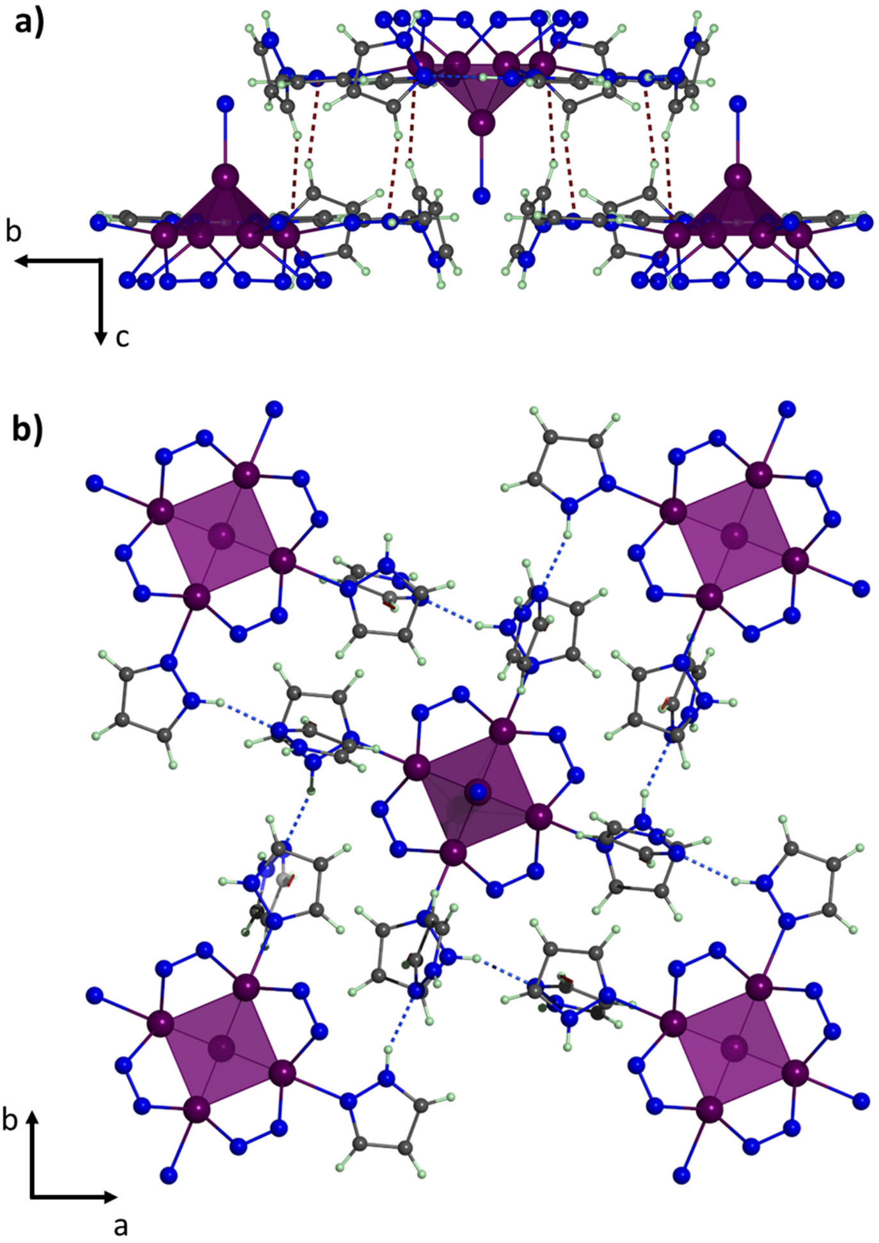

Figure S4. Packing in the crystal structure of **Mo_5red**. Hydrogen bonds and C-H···π stacking interactions between solvated pyrazole molecules and pzH-ligands along *a* (a) and *c* (b) axis. Color code: Mo – violet, Mo_5 – violet square pyramid, C – gray, N – blue, Br – cyan, H – light green, disordered C/N – green, C-H···π – red, N-H···N – blue, N-H···Br – cyan.

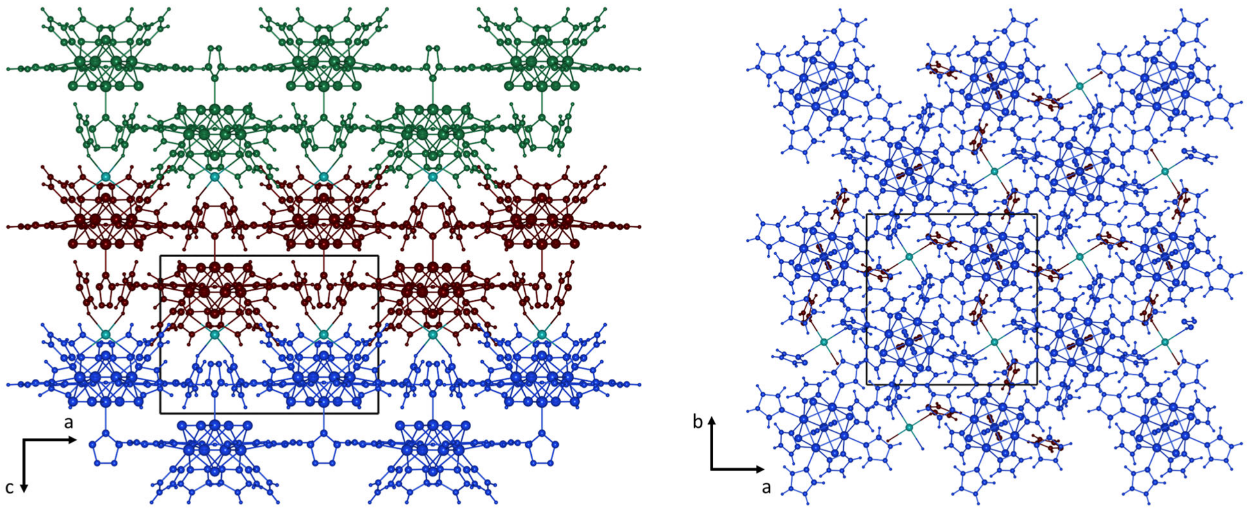

Figure S5. Packing of layers in the crystal structure of **Mo_5red**: side (left) and top (right) view.

Mo_5ox: packing in crystal structure and powder X-ray diffraction analysis

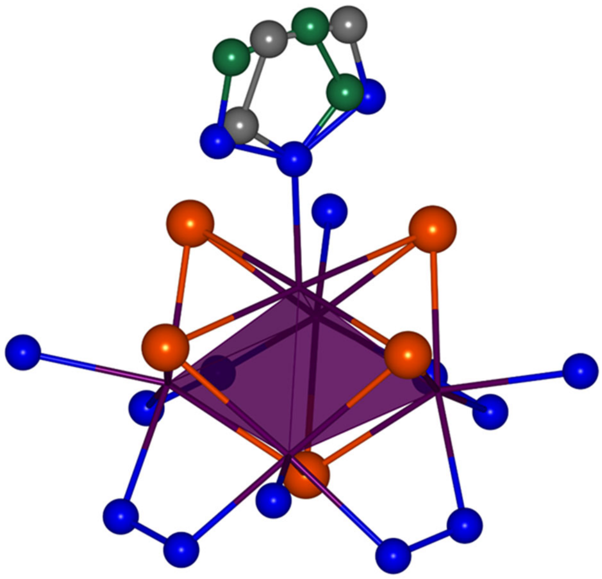

Figure S6. Disorder of axial pyrazole ligand over two positions in the crystal structure of **Mo_5ox**. Color code: Mo – violet, Mo_5 – violet square pyramid, C – gray/green, N – blue.

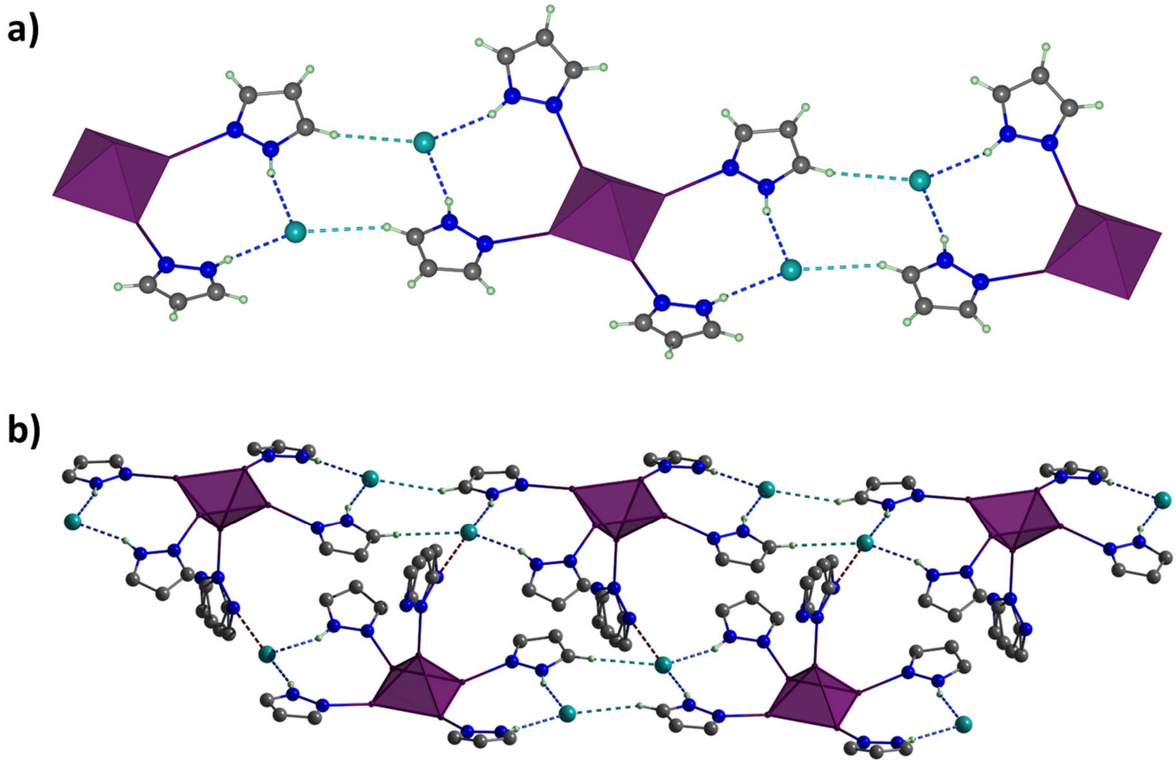

Figure S7. Packing in the crystal structure of **Mo_5ox**. a) Hydrogen bonds between terminal pyrazole ligands and Br forming infinite chains. b) Hydrogen bonds between bromine-ion and axial pyrazoles connecting chains. Color code: Mo – violet, Mo_5 – violet square pyramid, C – gray, N – blue, Br – cyan, H – light green, C-H···Br – cyan, N^{eq}-H···Br – blue, N^{ax}-H···Br – red.

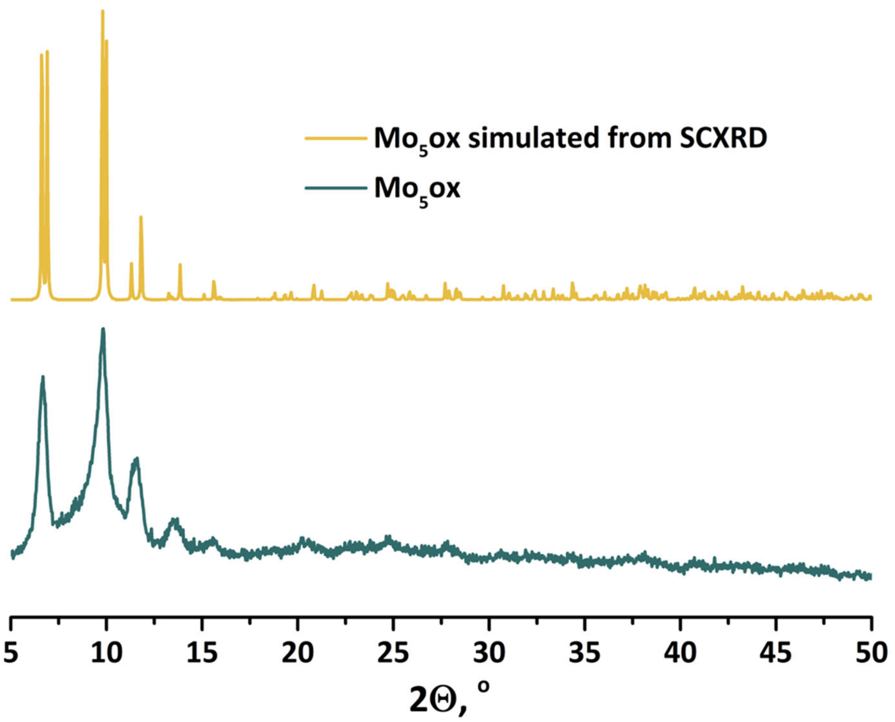

Figure S8. XRPD pattern of **Mo_5ox** in comparison with calculated one from the SCXRD data.

DFT calculations

Table S1. Optimized bond lengths (range | average) within the $[Mo_5(\mu_4\text{-}Se)(\mu_3\text{-}Se)_4(\mu\text{-}pz)_4pz_5]^{2+/1+}$ clusters at VWN/S12g/TZP level of theory and C_1 point symmetry.

Cluster / Bond	Mo_{eq}–Mo_{eq}	Mo_{eq}–Mo_{ap}	Mo_{eq}–(μ_3-Se)	Mo_{ap}–(μ_3-Se)	Mo_{eq}–(μ_4-Se)	Mo–N (apical pz)	Mo–N (μ-pz)
$[Mo_5(\mu_4\text{-}Se)(\mu_3\text{-}Se)_4(\mu\text{-}pz)_4pz_5]^{+}$	2.8430–2.8440<br>2.8435(6)	2.681–2.686<br>2.684(2)	2.530–2.550<br>2.540(8)	2.570–2.585<br>2.574(8)	2.558–2.561<br>2.560(1)	2.265–2.273<br>2.270(3)	2.209–2.215<br>2.212(2)
$[Mo_5(\mu_4\text{-}Se)(\mu_3\text{-}Se)_4(\mu\text{-}pz)_4pz_5]^{2+}$	2.862–2.872<br>2.867(5)	2.666–2.669<br>2.668(1)	2.514–2.531<br>2.523(7)	2.589–2.594<br>2.592(2)	2.5600–2.5610<br>2.5603(5)	2.245–2.262<br>2.256(7)	2.195–2.204<br>2.200(4)

Table S2. Orbital energies, symmetry of molecular orbitals and percentage contributions from the metal atoms, selenide ligands and pzH molecules for the $[\{Mo_5(\mu_4\text{-}Se)(\mu_3\text{-}Se)_4(\mu\text{-}pz)_4\}(pzH)_5]^{2+}$ cluster.

MO	Energy (eV)	Mo_5	μ_4-Se	μ_3-Se	μ-pz	Outer pz
352 (LUMO+2)	-2.384	75.43	–	24.57	–	–
351 (LUMO+1)	-3.762	82.12	2.36	8.76	6.76	–
350 (LUMO)	-4.306	67.76	1.29	30.95	–	–
349 (HOMO)	-6.345	79.84	4.98	10.59	3.11	1.48
348 (HOMO-1)	-6.411	87.11	5.64	1.42	5.83	–
347 (HOMO-2)	-6.684	73.71	–	15.65	–	10.64

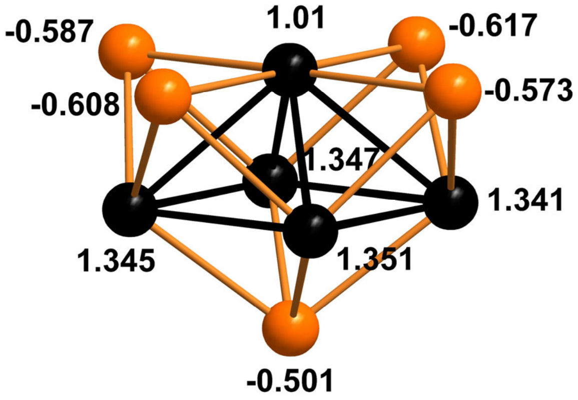

Figure S9. Bader charges on the atoms of the $\{Mo_5Se_5\}$ fragment of the $[\{Mo_5(\mu_4\text{-}Se)(\mu_3\text{-}Se)_4(\mu\text{-}pz)_4\}(pzH)_5]^{2+}$ cluster.

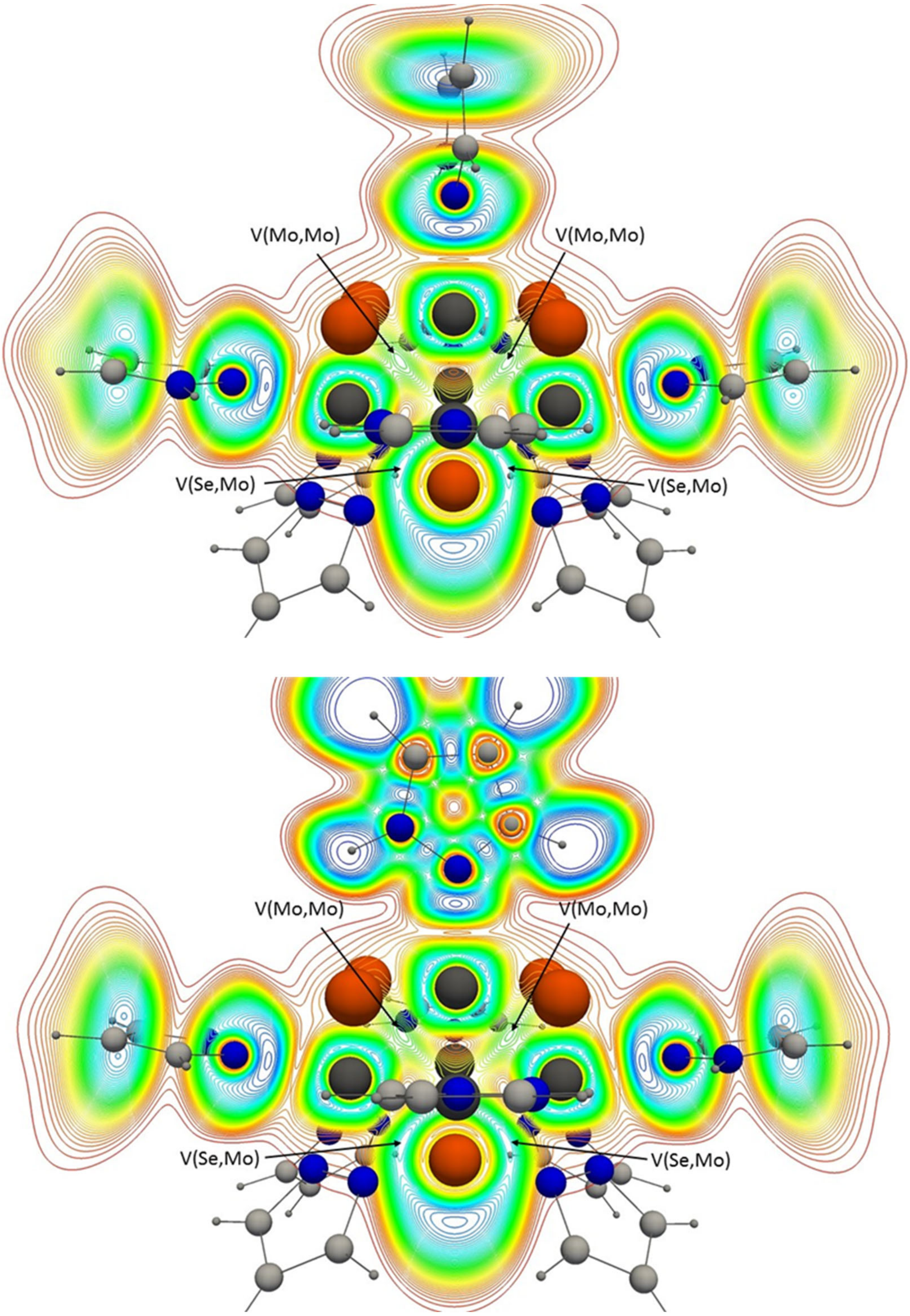

Figure S10. ELF maps for $[\{Mo_5(\mu_4\text{-}Se)(\mu_3\text{-}Se)_4(\mu\text{-}pz)_4\}(pzH)_5]^{2+}$ cluster (cutting planes Mo_{eq}–Mo_{ax}–Mo_{eq}–(μ_4-Se). Color code: Mo, dark gray; Se, orange; N, blue; C, H, light gray. V(Mo,Mo) and V(Se,Mo) are disynaptic basins between corresponding atoms.

XPS data

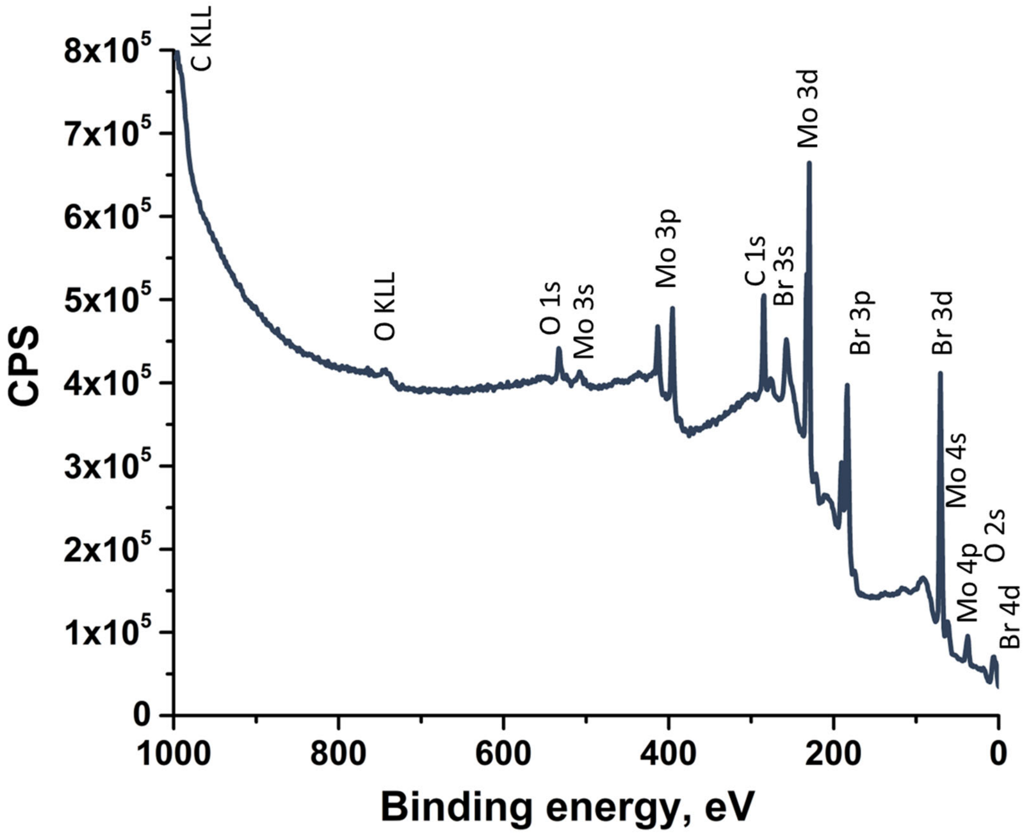

Figure S11. XPS survey spectrum of Mo_6Br_{12}.

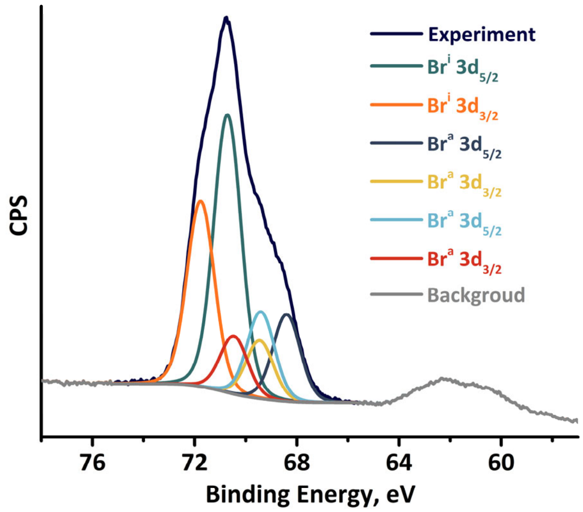

Figure S12. XPS spectrum showing Br 3d core level of Mo_6Br_{12}.

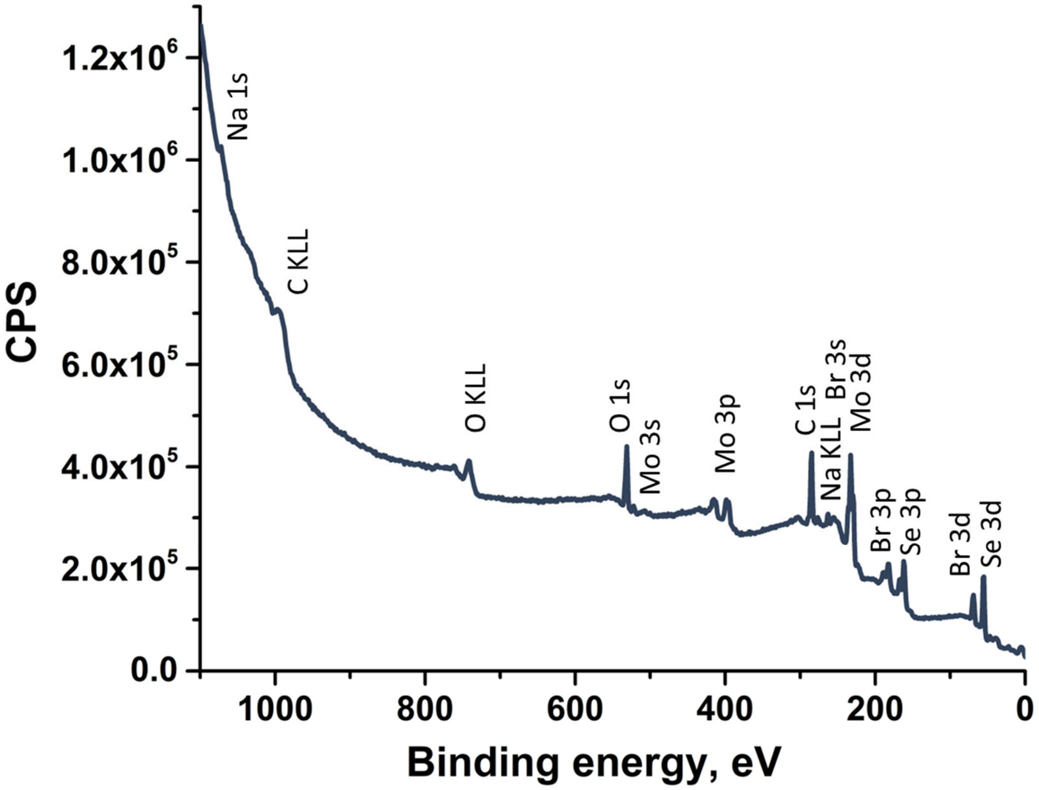

Figure S13. XPS survey spectrum of **Mo₆**.

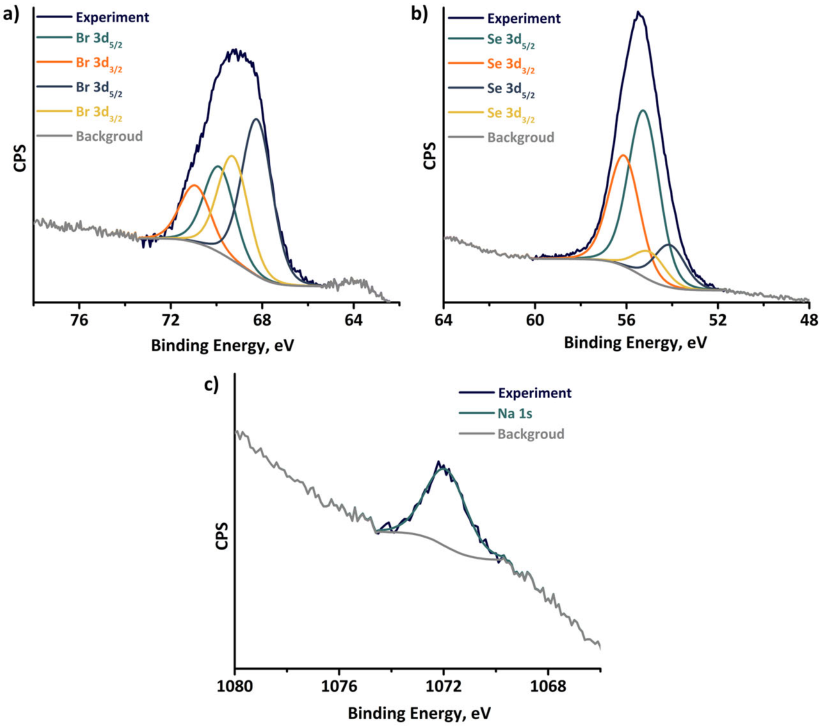

Figure S14. XPS spectra showing Br 3d (a), Se 3d (b) and Na 1s (c) core level of **Mo₆**.

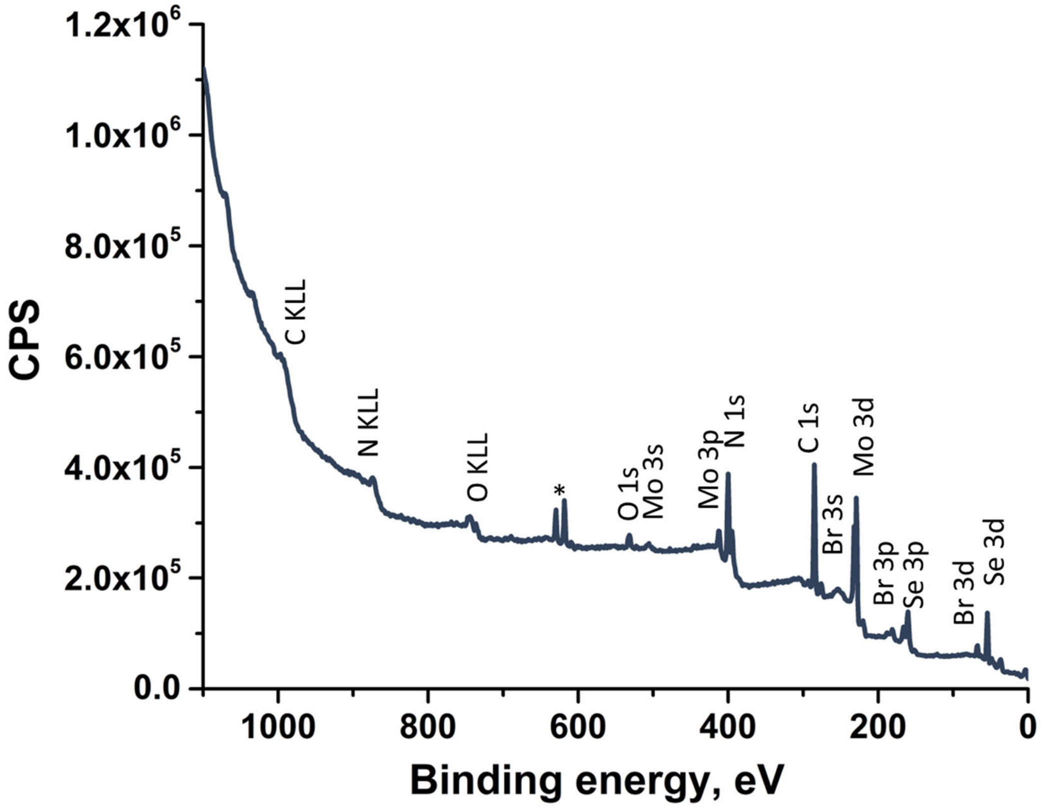

Figure S15. XPS survey spectrum of **Mo_5red**.

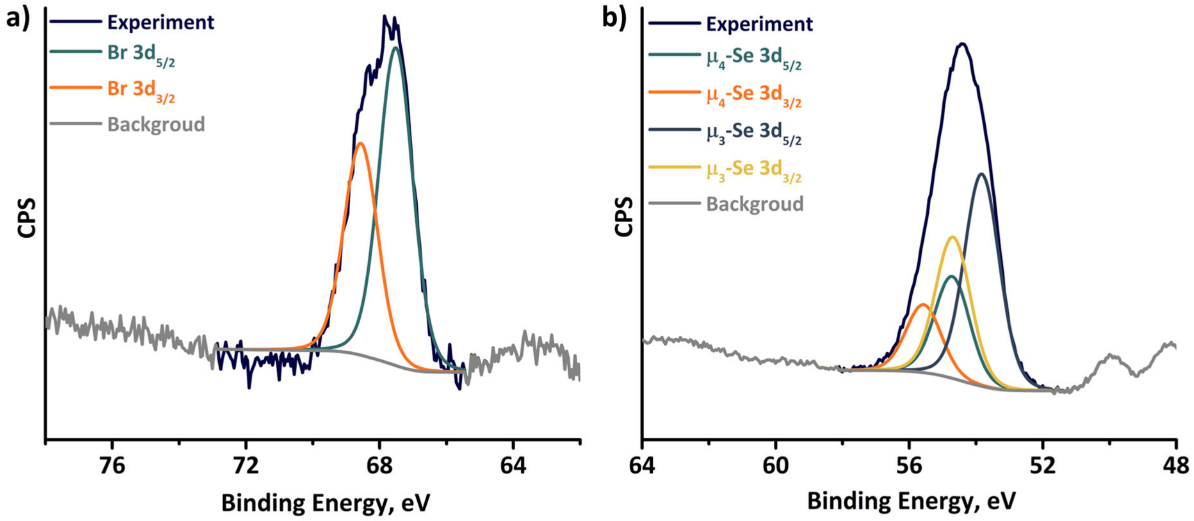

Figure S16. XPS spectra showing Br 3d (a) and Se 3d (b) core level of **Mo_5red**.

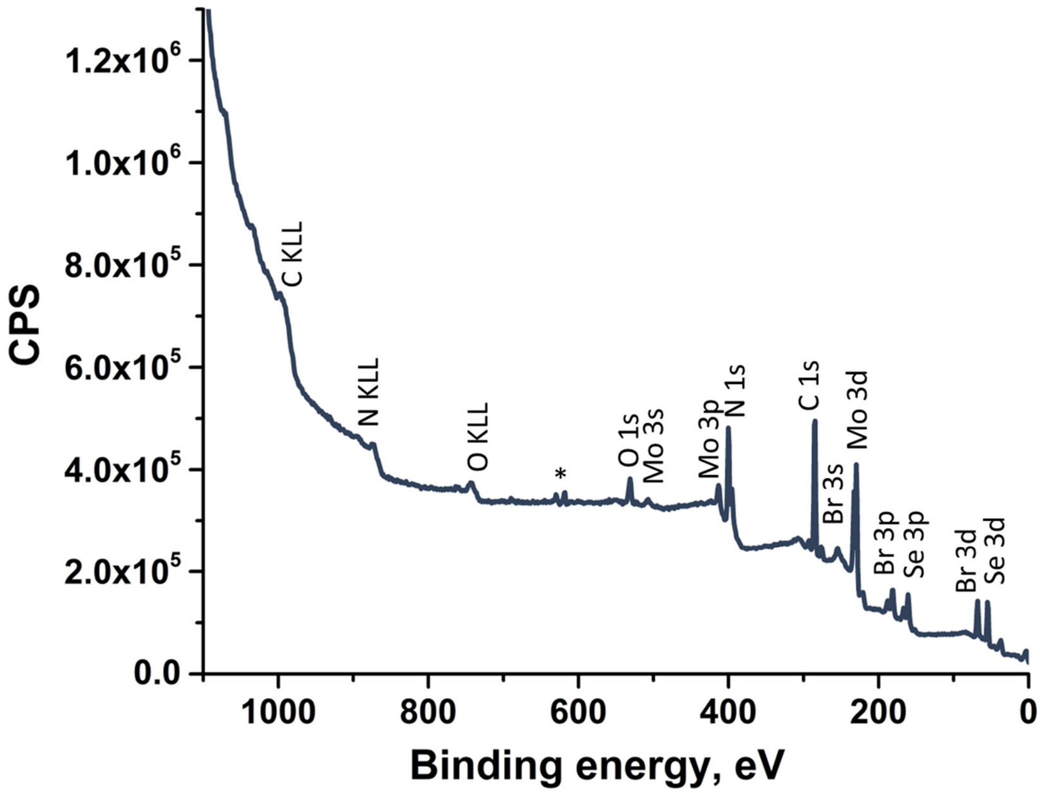

Figure S17. XPS survey spectrum of **Mo_5ox**.

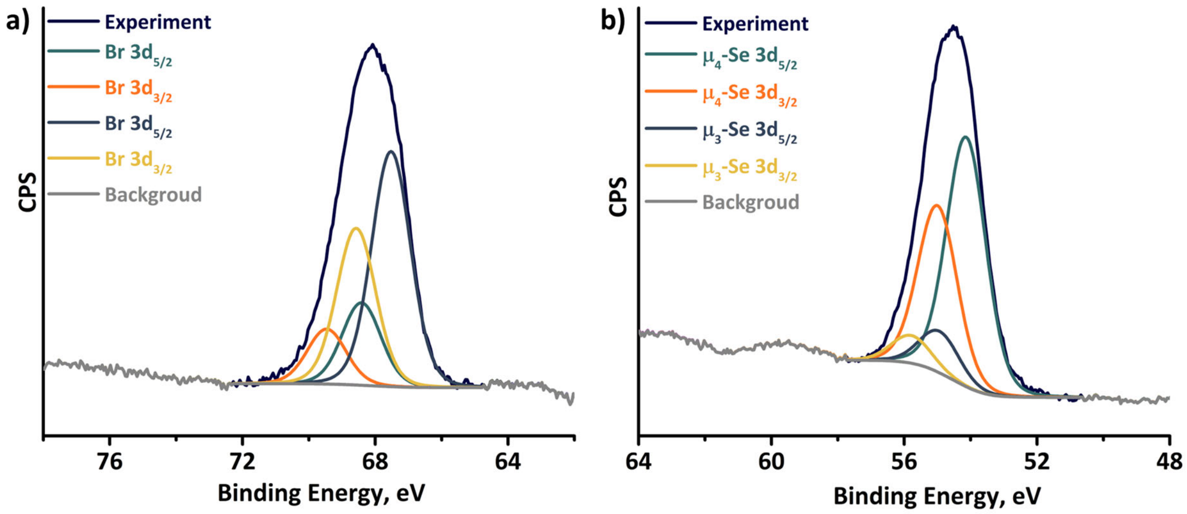

Figure S18. XPS spectra showing Br 3d (a) and Se 3d (b) core level of **Mo_5ox**.

Table S3. XPS binding energies (eV) in clusters obtained.

Compound	$Mo3d_{5/2,3/2}$ (%)	$Br3d_{5/2,3/2}$ (%)	$Se3d_{5/2,3/2}$ (%)	Na1s
Mo_6Br_{12}	229.8–232.9 (100%)	68.4-69.5 (19%)<br>69.4-70.5 (19%)<br>70.7-71.7 (62%)	–	–
**Mo_6**	229.2–232.4 (54%)<br>232.8–236.0 (46%)	68.2-69.3 (65%)<br>69.9-70.9 (35%)	54.1-55.0 (19%)<br>55.3-56.1 (81%)	1071.9
**Mo_5red**	228.9–232.1 (95%)<br>231.7–234.8 (4%)<br>233.3–236.4 (1%)	67.5-68.6	53.8-54.7 (66%)<br>54.7-55.6 (34%)	–
**Mo_5ox**	229.3–232.4 (78%)<br>230.7–233.9 (20%)<br>232.1–235.2 (2%)	67.5-68.6 (74%)<br>68.4-69.5 (26%)	54.1-55.0 (86%)<br>54.9-55.8 (14%)	–

NMR spectroscopy data

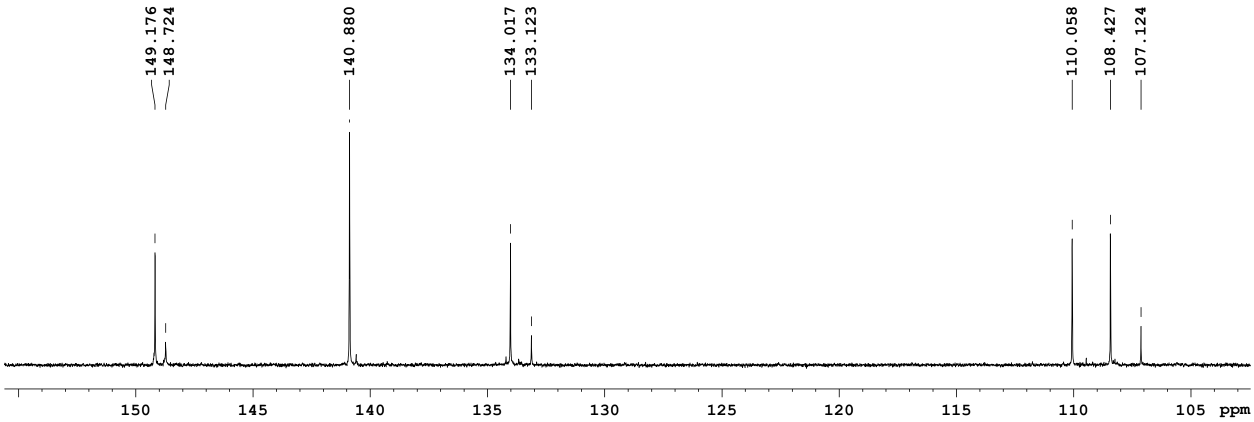

Figure S19. ^{13}C NMR spectrum of **Mo_5ox** in CD_3OD.

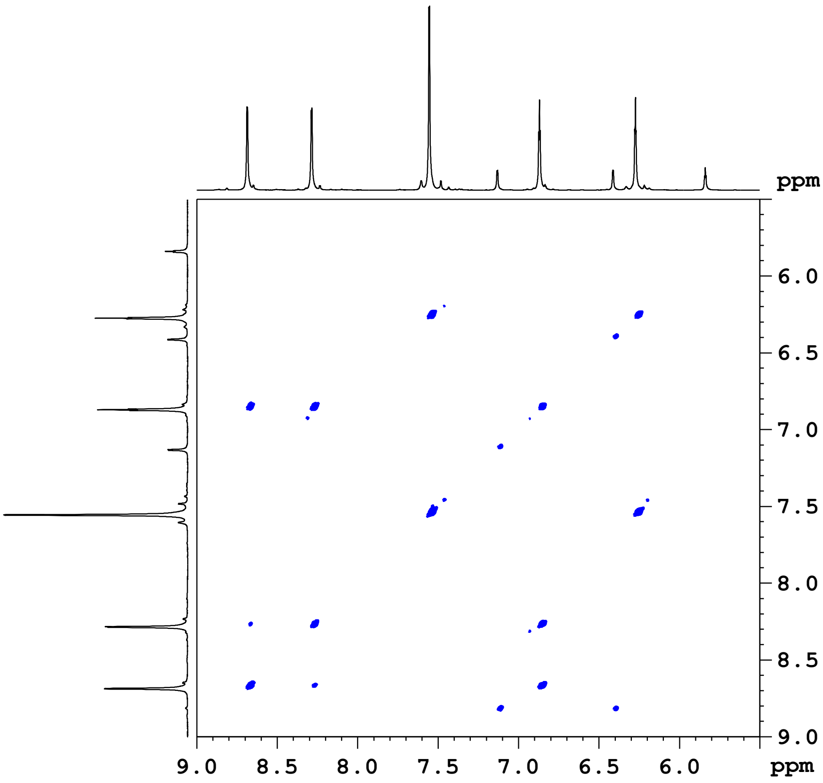

Figure S20. $^{1}H,^{1}H$-NMR correlation spectra of **Mo_5ox** in CD_3OD.

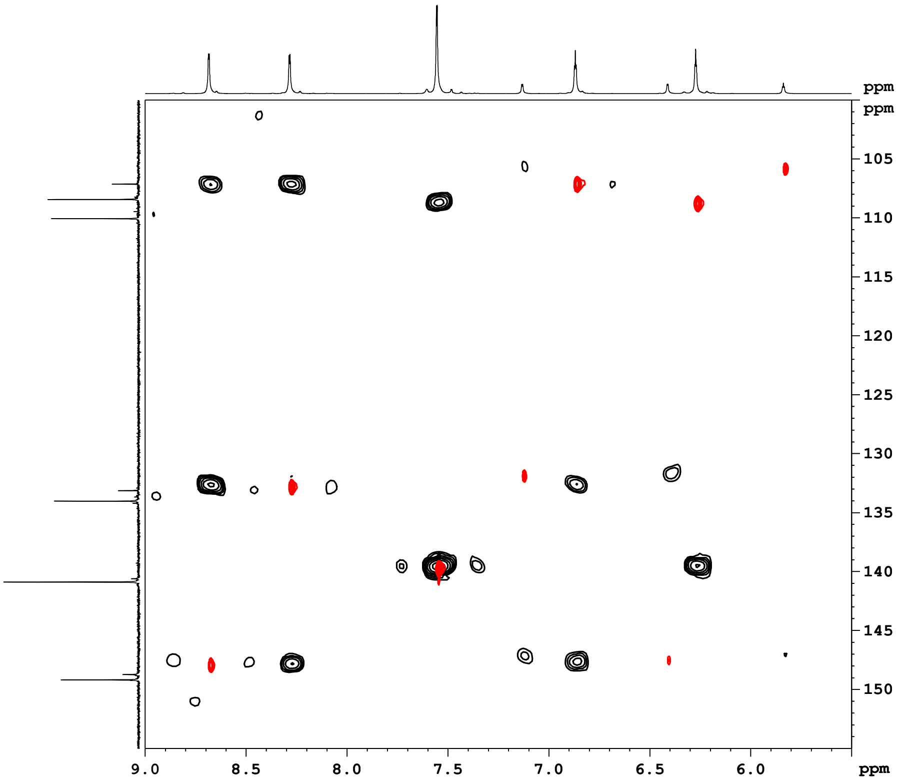

Figure S21. ^{1}H,^{13}C-NMR correlation spectra (HMBC – black, HSQC – red) of **Mo₅ox** in CD_3OD.

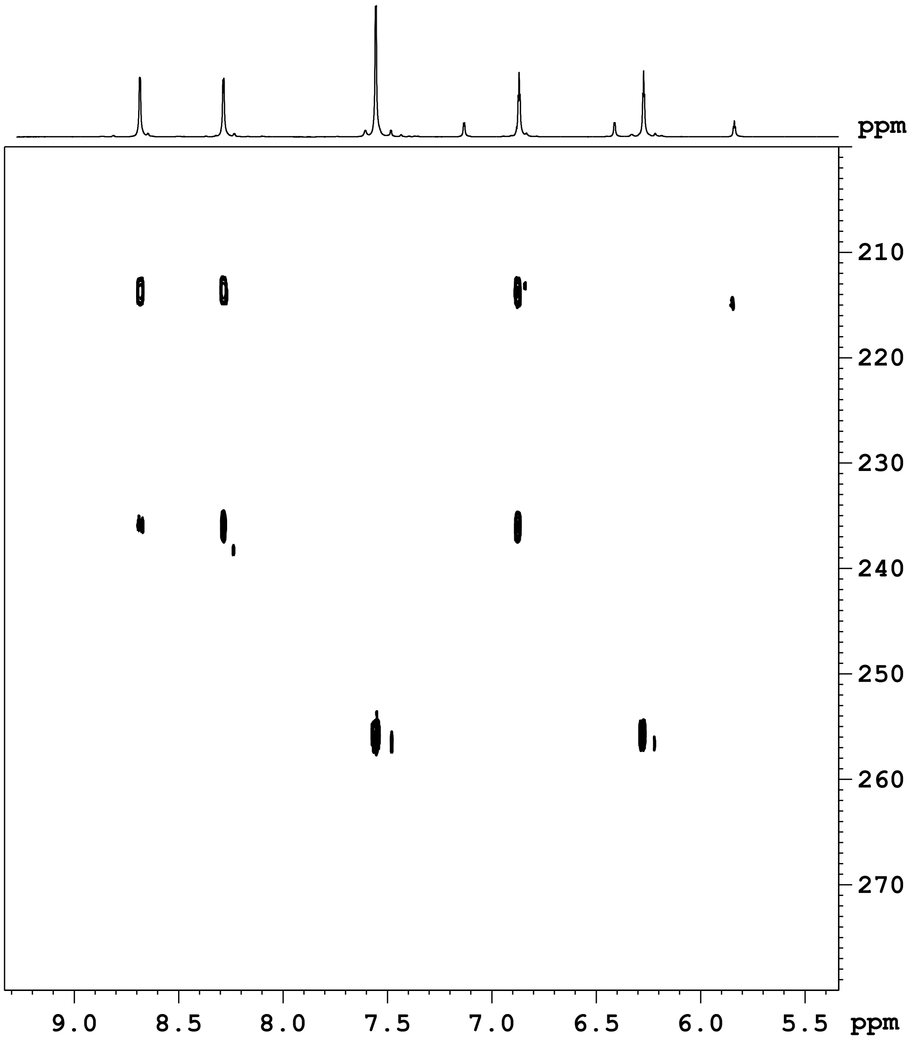

Figure S22. $^{1}H,^{15}N$-NMR HMBC spectrum of **Mo_5ox** in CD_3OD.

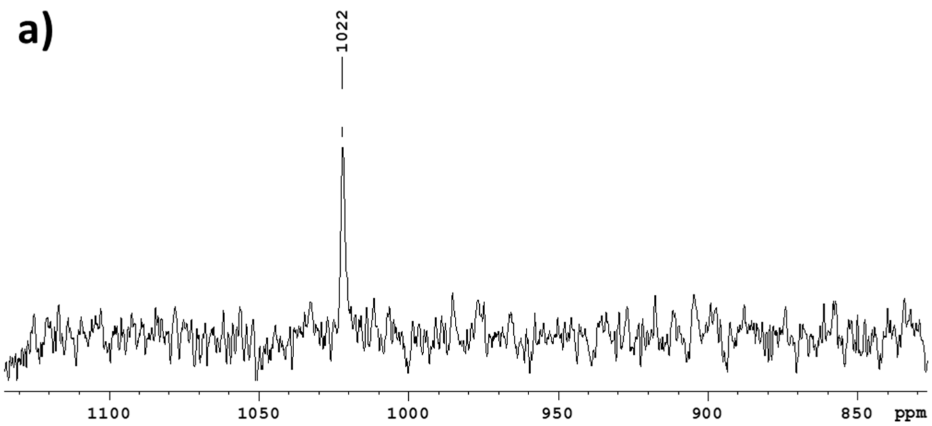

Figure S23. ^{77}Se NMR spectra of **Mo_5ox** in CD_3OD

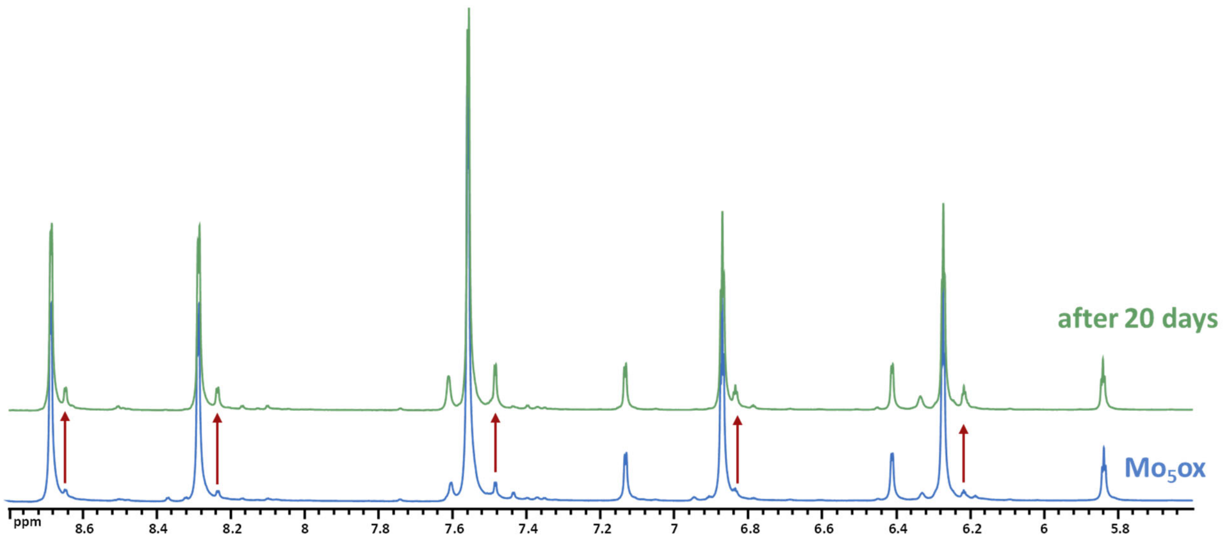

Figure S24. ^{1}H NMR spectra of **Mo_5ox** in CD_3OD in time. Red arrows indicate appearance of new signals of equatorial pyrazole and inner pyrazolate ligands.

Mass-spectrometry data

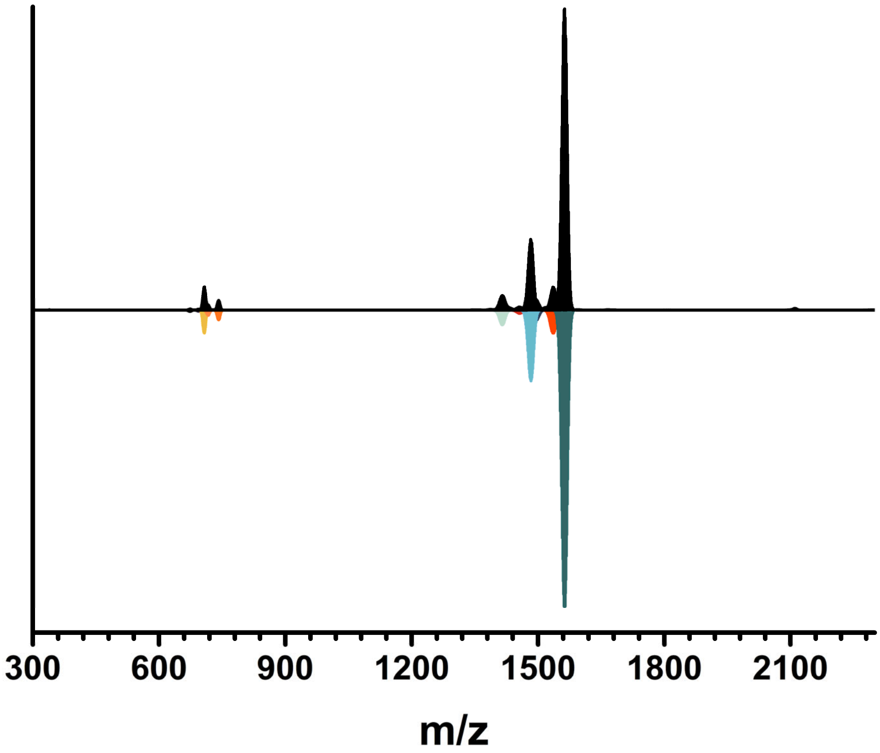

Figure S25. HR-ESI-MS spectrum of solution of **Mo_5ox** in acetonitrile (black) and simulations of cluster forms (colored).

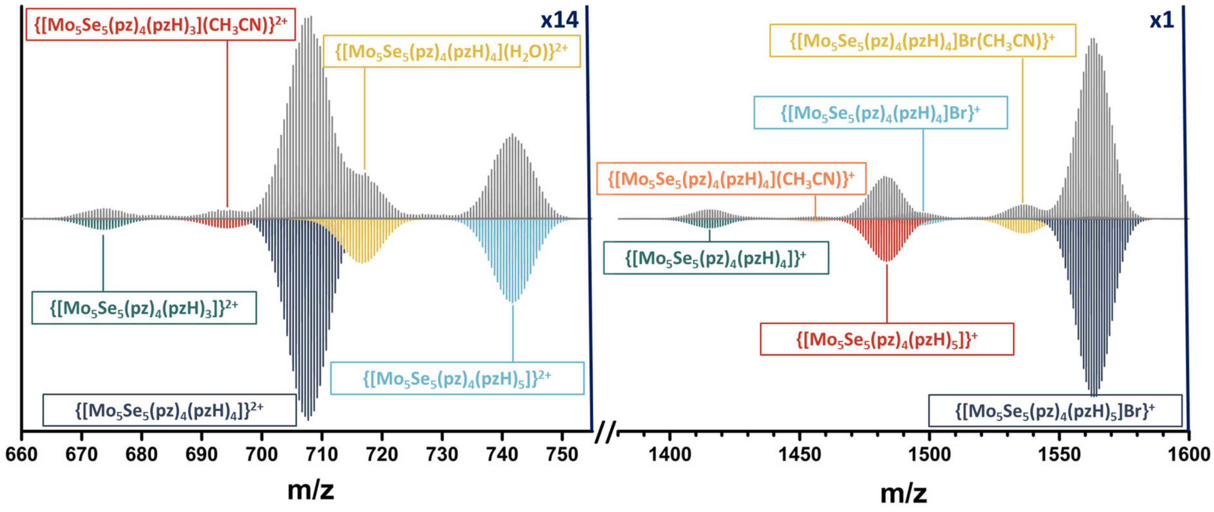

Figure S26. Fragment of HR-ESI-MS spectrum of solution of **Mo_5ox** in acetonitrile (gray) and simulations of cluster forms (colored).

EPR spectroscopy data

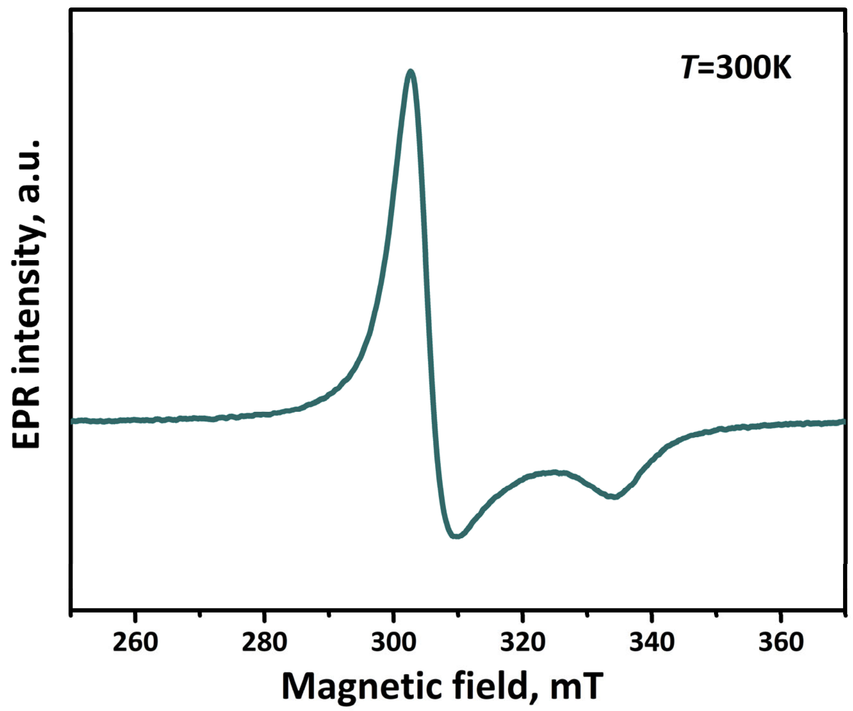

Figure S27. EPR spectrum of **Mo$_5$red** at 300 K. The *g*-tensor values are evaluated to be g_{xx} = g_{yy} = 2.19 and g_{zz} = 1.99 (g_{iso} = 2.12) changing slightly with temperature.

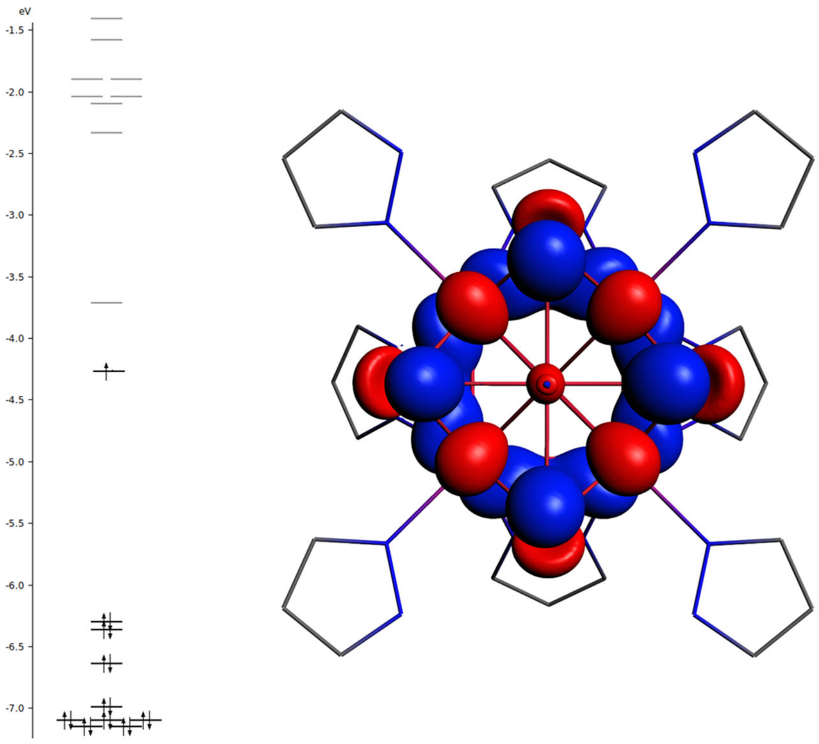

Figure S28. The HOMO orbital of the [{Mo$_5$(μ$_3$-Se)$_4$(μ$_4$-Se)(μ-pz)$_4$}(pzH)$_5$]$^+$ cation. The hydrogen atoms are omitted for clarity, the isosurface value is 0.035, view along the [001].

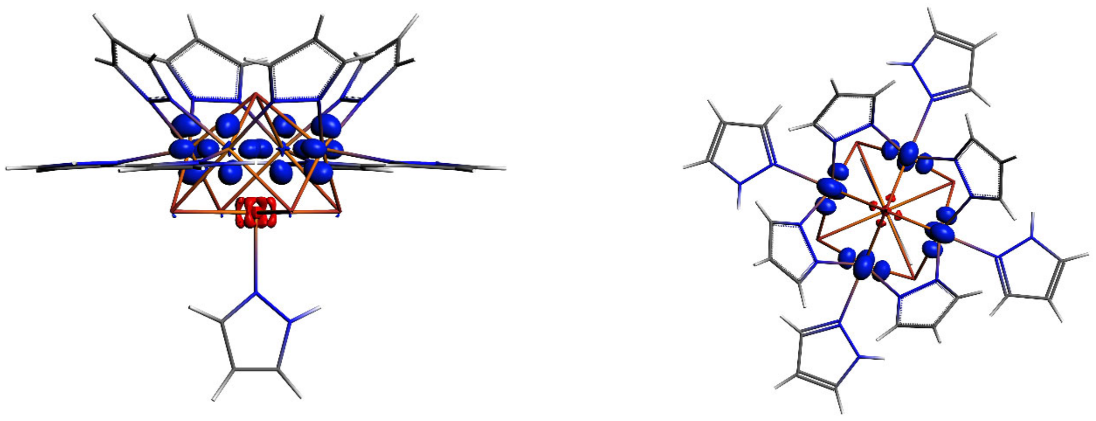

Figure S29. The plot of the spin density in the [{Mo_5(μ_3-Se)$_4$(μ_4-Se)(μ-pz)$_4$}(pzH)$_5$]$^+$ cation. The isosurface value 0.01, view along the [100] (left) and [001] (right). The spin density distribution in general corresponds to the *d*-orbitals of the basal Mo atoms.

Cyclic voltammetry data

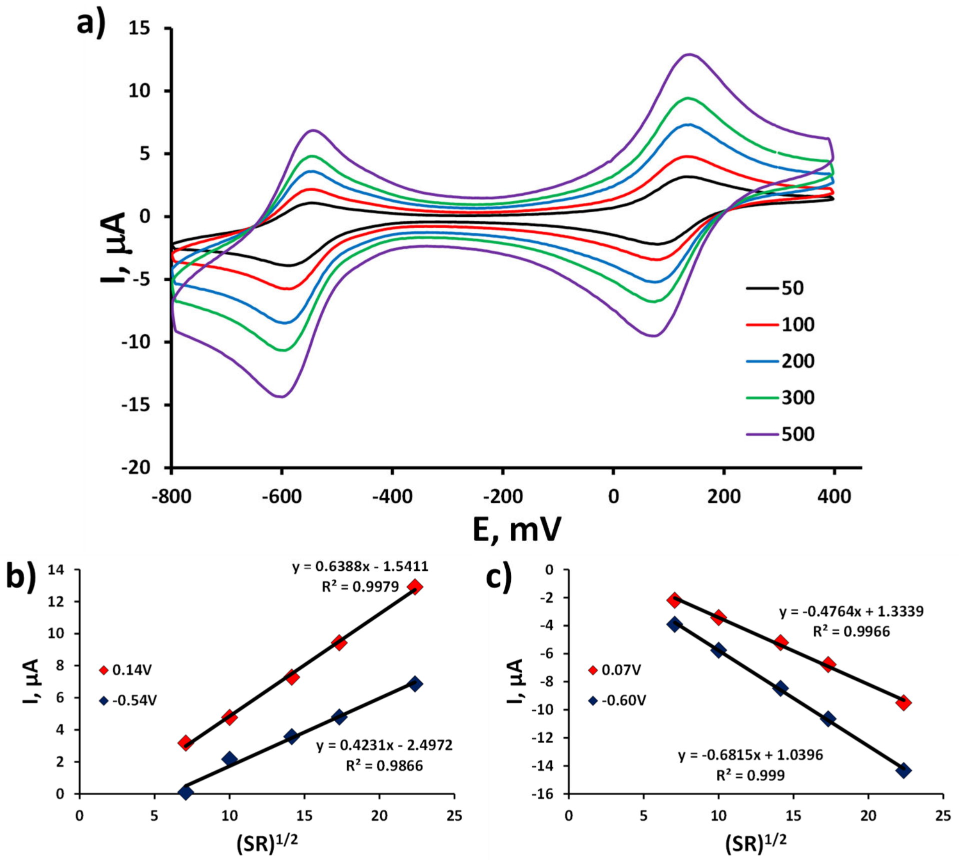

Figure S30. Cyclic voltammetry of the **Mo$_5$red** (0.5 mM) in 0.1 M Bu_4NClO_4 acetonitrile solution at different scan rates (a). Linear dependence of the anodic (b) and cathodic (c) peak current upon square root of the potential scan rate $\sqrt{SR}$;

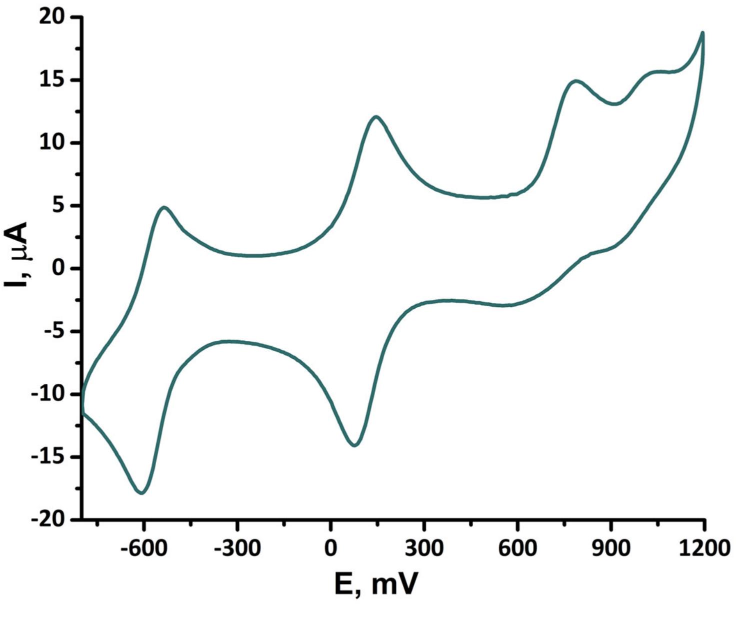

Figure S31. Cyclic voltammetry of the **Mo$_5$red** (0.5 mM) in 0.1 M Bu_4NClO_4 acetonitrile solution.

Characterization of compounds: IR spectra, TGA curves, UV-vis spectra and PXRD data

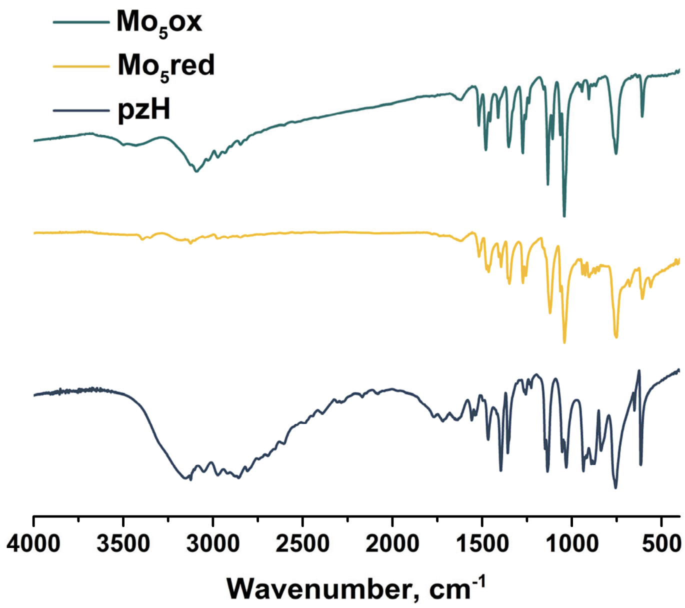

Figure S32. FTIR spectra of **Mo_5red** and **Mo_5ox** in comparison with pyrazole.

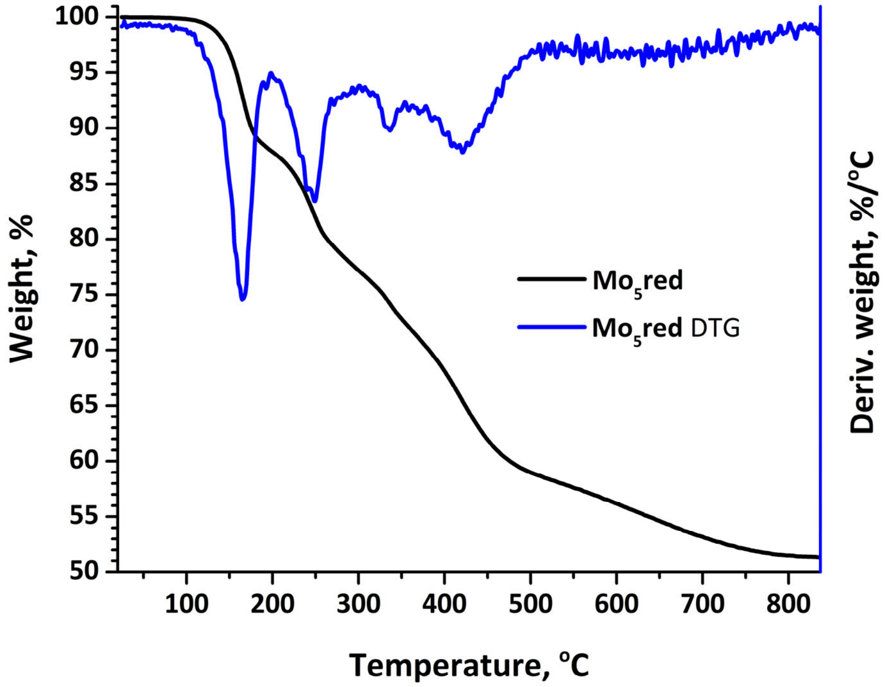

Figure S33. TGA and DTG curves of **Mo_5red**. Heating rates of 10 $^{o}C \cdot min^{-1}$.

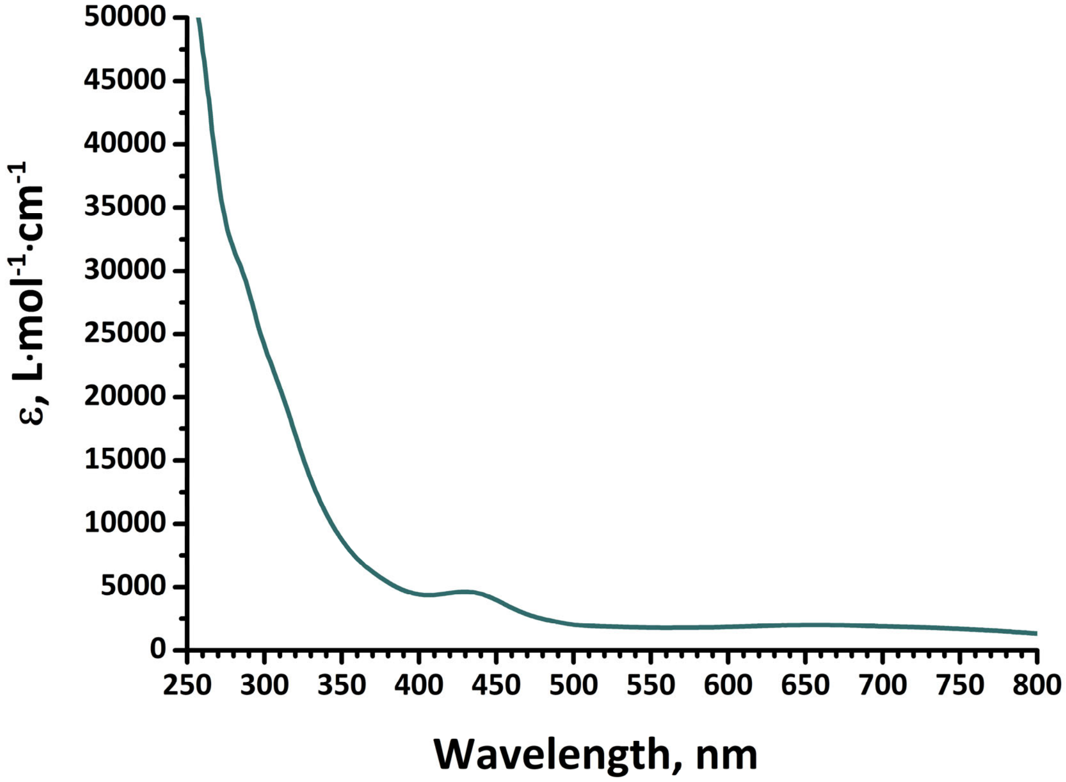

Figure S34. UV-vis spectrum of **Mo_5red** acetonitrile solution.

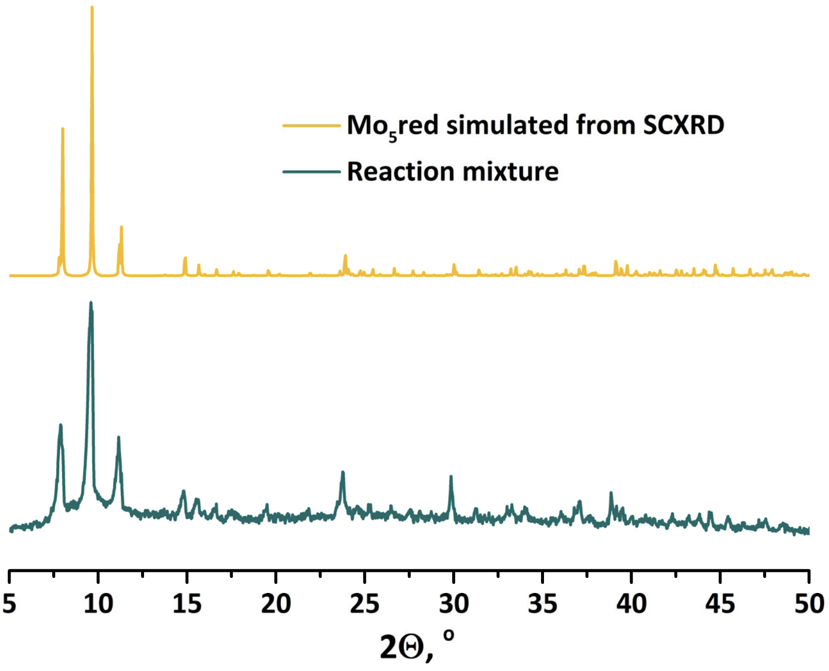

Figure S35. XRPD pattern of reaction mixture in comparison with calculated one from the SCXRD data for **Mo_5red**.

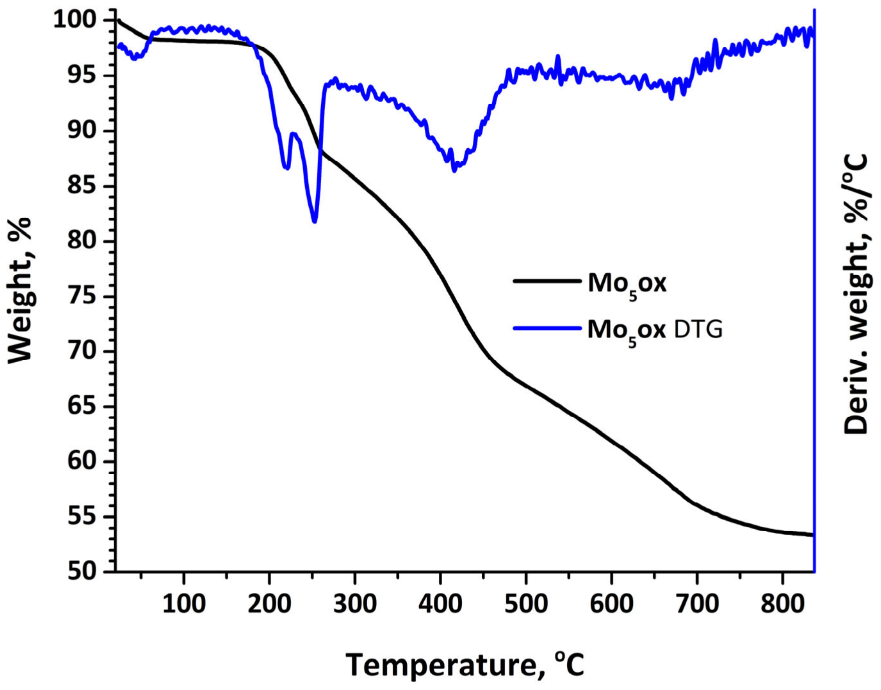

Figure S36. TGA and DTG curves of **Mo$_5$ox**. Heating rates of 10 $^oC \cdot min^{-1}$.

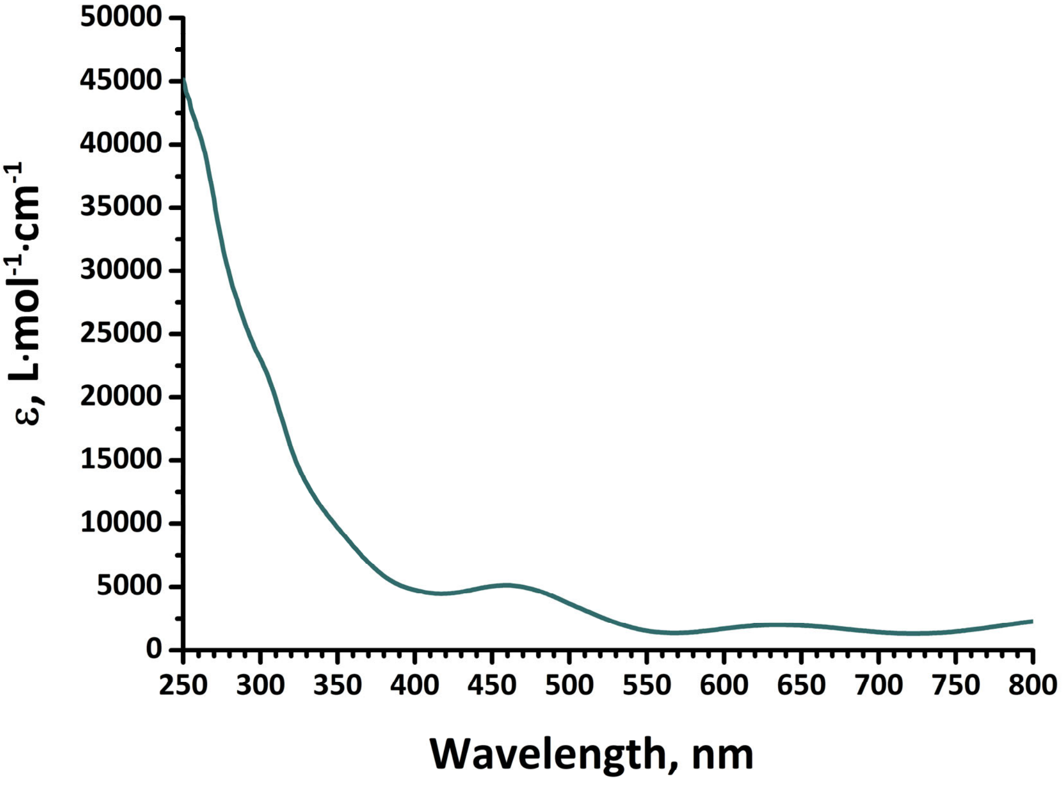

Figure S37. UV-vis spectrum of **Mo$_5$ox** acetonitrile solution.

Crystal structure data

Table S4. Selected crystallographic parameters of the single-crystal X-ray diffraction structural analysis for $[\{Mo_5(\mu_3\text{-}Se)^i{}_4(\mu_4\text{-}Se)^i(\mu\text{-}pz)^i{}_4\}(pzH)^a{}_5]Br$ 4pzH (**Mo5red**), $[\{Mo_5(\mu_3\text{-}Se)^i{}_4(\mu_4\text{-}Se)^i(\mu\text{-}pz)^i{}_4\}(pzH)^a{}_5]Br_2$ $2H_2O$ (**Mo5ox**).

Compound	**Mo5red**	**Mo5ox**
Empirical formula	$C_{39}H_{48}BrMo_5N_{26}Se_5$	$C_{27}H_{36}Br_2Mo_5N_{18}O_2Se_5$
Formula weight	1835.44	1679.06
Temperature, K	150(2)	150(2)
Crystal system	Tetragonal	Orthorhombic
Space group	*P*4/n	*C*222$_1$
a, Å	15.6443(4)	15.6320(10)
b, Å	15.6443(4)	25.5634(15)
c, Å	11.3058(2)	12.2766(8)
α, °	90	90
β, °	90	90
γ, °	90	90
V, Å^3	2767.03(15)	4905.8(5)
Z	2	4
ρ_{calc}, g/cm^3	2.203	2.273
μ, mm^{-1}	5.171	6.629
F (000)	1758	3160
Crystal size	0.22 × 0.15 × 0.12	0.21 × 0.15 × 0.12
2Θ range for data collection, °	1.801 to 28.298	2.255 to 27.256
Index ranges	$-20 \le h \le 19$<br>$-20 \le k \le 20$<br>$-15 \le l \le 9$	$-18 \le h \le 20$<br>$-32 \le k \le 32$<br>$-15 \le l \le 15$
Reflections collected	21000	20573
Independent reflections	3441 [R_{int} = 0.0251]	5450 [R_{int} = 0.0433]
Data/restraints/parameters	3441/0/183	5450/12/293
Goodness-of-fit on F^2	1.053	1.064
R_1 / wR_2(I > 2σ(I))	0.0197/0.0493	0.0331/0.0814
R_1 / wR_1 (all data)	0.0233/0.0506	0.0462/0.0852
$\Delta\rho_{max}/\Delta\rho_{min}$ (e·Å^{-3})	1.282/–1.362	0.913/–0.715
